# ROBO3s: a novel ROBO3 short isoform promoting breast cancer aggressiveness

**DOI:** 10.1038/s41419-022-05197-7

**Published:** 2022-09-03

**Authors:** Marcel Werner, Anna Dyas, Iwan Parfentev, Geske E. Schmidt, Iga K. Mieczkowska, Lukas C. Müller-Kirschbaum, Claudia Müller, Stefan Kalkhof, Oliver Reinhardt, Henning Urlaub, Frauke Alves, Julia Gallwas, Evangelos Prokakis, Florian Wegwitz

**Affiliations:** 1 Department of General, Visceral and Pediatric Surgery, University Medical Center Göttingen, Göttingen, Germany; 2 Chromosome Dynamics and Genome Stability, Institute of Epigenetics and Stem Cells, Helmholtz Zentrum München, Munich, Germany; 3 International Max-Planck Research School for Molecular Biology, Göttingen, Germany; 4 Early Cancer Institute, University of Cambridge, Department of Oncology, Hutchison Research Centre, Box 197 Cambridge Biomedical Campus, Cambridge, Germany; 5 Bioanalytical Mass Spectrometry group, Max Planck Institute for Multidisciplinary Sciences, Göttingen, Germany; 6 Department of Gastroenterology, Gastrointestinal Oncology and Endocrinology, University Medical Center Göttingen, Göttingen, Germany; 7 Department of Preclinical Development and Validation, Fraunhofer Institute for Cell Therapy and Immunology, Leipzig, Germany; 8 Translational Molecular Imaging, Max-Planck Institute for Multidisciplinary Sciences, Göttingen, Germany; 9 Bioanalytics, Institute of Clinical Chemistry, University Medical Center Göttingen, Göttingen, Germany; 10 Department of Hematology and Medical Oncology, University Medicine Goettingen, Göttingen, Germany; 11 Department of Gynecology and Obstetrics, University Medical Center Göttingen, Göttingen, Germany

**Keywords:** Breast cancer, Cancer stem cells, Oncogenes, Tumour biomarkers

## Abstract

Basal-like breast cancer (BLBC) is a highly aggressive breast cancer subtype frequently associated with poor prognosis. Due to the scarcity of targeted treatment options, conventional cytotoxic chemotherapies frequently remain the standard of care. Unfortunately, their efficacy is limited as BLBC malignancies rapidly develop resistant phenotypes. Using transcriptomic and proteomic approaches in human and murine BLBC cells, we aimed to elucidate the molecular mechanisms underlying the acquisition of aggressive and chemotherapy-resistant phenotypes in these mammary tumors. Specifically, we identified and characterized a novel short isoform of Roundabout Guidance Receptor 3 (*ROBO3s*), upregulated in BLBC in response to chemotherapy and encoding for a protein variant lacking the transmembrane domain. We established an important role for the *ROBO3s* isoform, mediating cancer stem cell properties by stimulating the Hippo-YAP signaling pathway, and thus driving resistance of BLBC cells to cytotoxic drugs. By uncovering the conservation of ROBO3s expression across multiple cancer types, as well as its association with reduced BLBC-patient survival, we emphasize its potential as a prognostic marker and identify a novel attractive target for anti-cancer drug development.

## Introduction

With over 2 million new cases worldwide every year, breast cancer (BC) is by far the most frequent malignancy in women [1]. Advances in early detection, classification, and targeted therapies have led to a tremendous improvement in patient survival in the last decades. Tumors with limited size and low metastatic load can be surgically removed and generally correlate with a good prognosis [2]. Despite this, approximately 10–15% of BC patients develop metastatic tumors within three years of diagnosis, which is the major cause of death for BC [3]. Great research efforts over the last decades have led to the development of targeted therapies such as small molecule inhibitors or monoclonal antibodies. However, basal-like breast cancer (BLBC) and triple-negative breast cancers (TNBC), two largely overlapping BC subtypes, lack the expression of currently druggable molecular targets [4, 5]. The therapeutic options to treat these malignancies are, therefore, very limited and mostly restricted to conventional chemotherapies [6]. Additionally, BLBC lesions rapidly develop resistance and frequently develop metastases [7–9]. These complications create an urgent need for the development of new strategies to improve the treatment efficacy for this particular disease subtype.

We previously developed and characterized WAP-T mice as a model to study the biology of basal-like mammary carcinoma [10, 11]. WAP-T tumor cells are remarkably plastic in vivo and in vitro, which accurately recapitulates the biology of metastasizing BLBC [12–15]. Recently, we treated WAP-T tumors with a combination of Cyclophosphamide/Adriamycin/5-fluorouracil (short CAF) and observed that a single cycle of this treatment is not able to eradicate the disease [16]. Notably, recurrences recapitulated the human situation, showing increased aggressiveness and dissemination properties, pronounced epithelial-to-mesenchymal transition (EMT), and cancer stem cell (CSC) traits [17, 18].

In the present study, we sought to investigate the mechanisms allowing BLBCs to escape cytotoxic treatments. Taking advantage of high-throughput transcriptome analyses, we identified Roundabout Homolog 3 (*Robo3*) as one of the most strongly induced genes in WAP-T cells surviving CAF treatment in vitro and in vivo. ROBO3 is one of the four members of the Roundabout (ROBO) receptors family that is involved in axon guidance pathways [19, 20]. The binding of ROBO receptors to their ligands, the SLIT proteins, generally induces a chemo-repulsive behavior [21]. As a divergent member of its family, ROBO3 only shows a weak capacity to interact with SLITs [19]. ROBO3 is most highly expressed during development in the central nervous system, and its expression is maintained in the adult nervous and sensory organ systems [22, 23]. Notably, ROBO3 has a major role in commissural axon midline crossing, where it controls the switch between chemo-attraction and chemo-repulsion [24]. Besides their well-characterized role in the nervous system, members of the SLIT/ROBO axon guidance pathway have also been implicated in the regulation of proliferation, homeostasis, and migration processes in various other organs [25, 26]. Moreover, high-throughput genomic analyses by the International Cancer Genome Consortium (ICGC.org) revealed that frequent genetic and epigenetic alterations in pancreatic cancer result in aberrant expression of axon guidance signaling members [27, 28]. However, studies of ROBO/SLIT signaling in cancers are scarce and the implication of ROBO3 in mammary carcinoma is lacking so far. In the present study, we describe for the first time a novel *ROBO3* short isoform (*ROBO3s*) supporting the survival of BLBC cells upon conventional cytotoxic chemotherapy with a promising potential as a diagnostic factor or therapeutic target.

## Materials and methods

### Cell culture

All cell lines were cultivated in the appropriate culture medium supplemented with 10% FBS, 100 units/mL penicillin and 100 µg/mL streptomycin at 37 °C and 5% CO_2_, as listed in Table S1.

### Animal experiments

WAP-T animals were bred under specific pathogen free conditions and treated according to German regulations for animal experiments (Niedersächsisches Landesamt für Verbraucherschutz und Lebensmittelsicherheit, LAVES, authorization 33.19-42502-04-16/1621). Briefly, 1 × 10^6^ H8N8 cells were injected into the right abdominal mammary gland of 8 to 10 weeks old virgin in WAP-T-NP8 mice. Once growing tumors reached 500 mm³, animals were treated with one dose of 100 mg/kg body weight cyclophosphamide (Endoxan, Baxter, Deerfield, IL, US), 5 mg/kg BW doxorubicin (Cell Pharm, Hannover, DE) and 100 mg/kg BW 5-FU (Medac, Wedel, DE). Tumors were dissected at day 6 post-treatment.

### Primary TNBC cell culture

The triple-negative breast cancer samples were obtained from chemotherapy treated patients, with their informed consent, at the Clinic Braunschweig upon acceptance of the Ärtzekammer Niedersachsen (authorization Grae/231/2018). Patient biopsies were placed in a sterile 2 ml tube containing cell culture medium (DMEM:F12 (PanBiotech, DE) with 20% FBS (Merck, DE), 0.023 U/ml Insulin, 0.5 µg/ml Hydrocortison and 10 ng/ml hEGF (all from Sigma Aldrich, DE)) at 4 °C during transport. Upon arrival, biopsies were minced with a sterile scalpel in 1–3 mm^3^ sections and cultured into a 6-well plate. The experiments were performed on expanded primary tumor cells at passages lower than p5.

### Chemotherapy treatment

Chemotherapeutic drugs were obtained at the pharmacy of the University Medical Center Göttingen. Treatment concentrations are provided in Table S2.

### Functional assays

Functional assays assessing proliferation and migration of BLBC cells were performed 24–72 h after siRNA transfection. Detailed descriptions of the individual assays are available in the supplementary methods.

### Protein analysis

Protein extraction and quantification were performed according to standard protocols. Samples were subsequently analyzed by western blot or mass spectrometry. Detailed descriptions of both methods including mass spectrometry data analysis are provided in the supplements. Full and uncropped western blots are available in the supplemental data.

### Gene expression analysis by qRT-PCR

RNA was extracted using QIAzol® (Qiagen) and reverse transcribed into cDNA with the M-MuLV reverse transcriptase (New England Biolabs). Relative gene expression was assessed by SYBR green-based qRT-PCR using a CFX Biorad system (Bio-Rad Laboratories). Detailed qRT-PCR protocol and primer list are available in the supplemental methods and Table S4, respectively.

### RNA library preparation

RNA samples were prepared using the TruSeq RNA Library Prep Kit v2 (Illumina) according to the Sample Preparation Guide (Illumina). Detailed protocols are provided in the supplements.

### mRNA sequencing analysis

RNA sequencing raw data were processed in the GALAXY environment [29] provided by the GWDG (https://galaxy.gwdg.de/). Enrichr (http://amp.pharm.mssm.edu/Enrichr/) and Gene Set Enrichment Analysis (Broad Institute) were used to identify gene signatures enriched in the different experimental conditions. Detailed information for sequencing data processing is available in the supplements.

### Analysis of publicly available datasets

Publicly available mRNA-seq and ChIP-seq datasets (https://www.ebi.ac.uk/) were processed in the Galaxy environment provided by the GWDG, as described in supplemental methods and Table S3. Patient data from the TCGA-BRCA dataset (downloaded at the https://xenabrowser.net/) were filtered along with the PAM50 basal-like subtype. Detailed data processing steps are described in the supplemental methods. Bigwig files of normal (GTex) and breast cancer tissues (TCGA-BRCA) were downloaded using the recount3 tool (http://rna.recount.bio/) [30]. Patient survival analysis for ROBO3^low^ and ROBO3^high^-expressing BLBC patients in kmplot.com were performed with following parameters: Subtype: StGallen-basal, systemic treatments: endocrine therapy-exclude, chemotherapy-any, split patients by-autoselect best cutoff.

### Data visualization, statistical analysis and figures generation

Plots were generated with R (v4.0.2) in the RStudio environment (v1.1.383) or with Graphpad Prism v.8.0.1. Error bars indicate the standard error mean (SEM), results of statistical tests are depicted as the following: *p < 0.05, **p < 0.01, ***p < 0.001.

### Reporting Summary

Further information on research design is available in the Nature Research Reporting Summary linked to this article.

## Results

### Murine BLBC cells strongly upregulate an uncharacterized short isoform of Robo3 during conventional chemotherapy survival

**Fig. 1:**
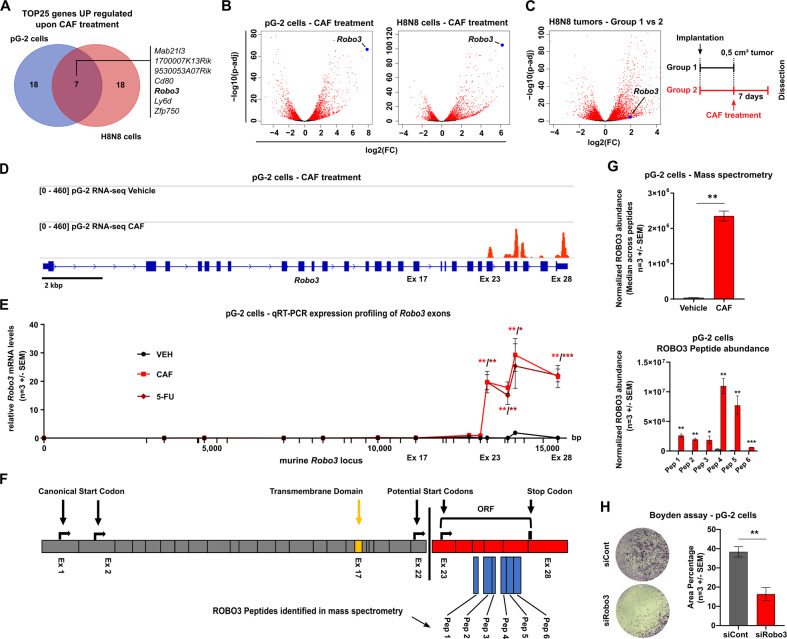
Murine BLBC cells strongly upregulate an uncharacterized short isoform of Robo3 during conventional chemotherapy survival. **A** Venn diagram of the top 25 most strongly upregulated genes in pG-2 and H8N8 cell lines after 48 h CAF chemotherapy (log2FC > 1 and padj < 0.05 relative to vehicle treatment). **B** Log2 fold change (Log2FC) gene expression volcano plots for pG-2 and H8N8 cell lines treated with 1:32 CAF chemotherapy for 48 h relative to vehicle treatment. Strongly upregulated *Robo3* labeled (blue). Genes exhibiting a significant regulation with an adjusted p-value (padj) of < 0.05 are highlighted (red). **C** Treatment scheme for groups 1 and 2 of WAP-T mice injected with H8N8 for tumor induction and subsequent CAF chemotherapy treatment (right panel). Log2FC gene expression volcano plots comparing groups 1 and 2 (left panel). Upregulated *Robo3* labeled (blue). Genes exhibiting a significant regulation with an adjusted padj of < 0.05 are highlighted (red). **D** Integrated Genome Browser (IGV) tracks of mRNA-seq of pG-2 cells treated with CAF or vehicle for 48 h. Reads corresponding to *Robo3* transcription (red) range from exon 23 to exon 28 upon CAF treatment but are absent in vehicle controls. **E** Overview of qRT-PCRs with primers covering the entire *Robo3* gene supports the restriction of *Robo3* transcription to exons 23–28 in pG-2 cells after CAF or 5-FU treatment for 48 h relative to vehicle. **F** Schematic depiction of potentially translated (red) and non-translated (grey) *Robo3* exons. Analysis of open reading frames (ORF) shows a possible start codon for translation with exon 23. ROBO3 peptide sequences identified by mass spectrometry are mapped against the amino acid sequence (blue). Canonical and newly predicted ORFs of ROBO3 terminate on an identical stop codon. The coding sequence for the ROBO3 transmembrane region is absent from the novel isoform (yellow). **G** Protein abundance of murine ROBO3 after CAF treatment in comparison to a control. The area under the curve of extracted fragment ion chromatograms was summed up and averaged for six peptides unique for ROBO3s. **H** Representative crystal violet stainings of a migration assay (Boyden chamber) on pG-2 cells treated either with siRobo3 or siRNA control (left panel). Quantification of migratory cells (area fraction) for each condition (right panel). **E, G**, **H:** Error bars represent mean ± SEM of three biological replicates, unpaired t-test: *p < 0.05; **p < 0.01; ***p < 0.001.

To understand the transcriptional changes that occur in BLBC cells during conventional CAF chemotherapy resistance, we performed mRNA-sequencing (mRNA-seq) in two previously characterized chemotherapy naïve cell lines, pG-2 and H8N8, established from WAP-T tumors [12, 15, 17]. Differential gene expression analysis identified 782 genes commonly upregulated and 117 genes commonly downregulated (Fig. S1A). Comparing the 25 most strongly upregulated genes upon CAF chemotherapy, 7 genes were shared across both cell lines (Fig. 1A). The upregulation of *Robo3* particularly drew our attention because of its dramatic and consistent upregulation across experimental systems, and its potential implication in cancer cell proliferation and metastasis [31] (Fig. 1A, B). *Robo3* was also upregulated upon CAF treatment of H8N8 cells orthotopically implanted into syngeneic WAP-T mice, in vivo (Fig. 1C).

Surprisingly, the read coverage of *Robo3* in the mRNA-seq data exclusively mapped to a region spanning from exon 23 to 28 (Fig. 1D). This was validated by qRT-PCRs for *Robo3* expression, which consistently only generated a measurable signal in the 3’ end region spanning from exon 23 to 28 (Fig. 1E, Fig. S1B and Table S4). To exclude the possibility that our mRNA-seq alignment failed to correctly map 5’-specific *Robo3* reads, we compared our data to publicly available mRNA-seq data of mouse brains. We observed a read coverage across all exons, confirming the expected expression of full-length *Robo3* in the nervous system (Fig. S1C).

To investigate if the expression of *Robo3* short transcript (referred to as *Robo3s* in this manuscript) is induced by an alternative promoter, we evaluated the occupancy changes of histone 3 at lysine 27 actylation (H3K27ac), marking active regulatory regions, at the *Robo3* locus. Previously published chromatin immunoprecipitation sequencing (ChIP-seq) data from our group revealed no detectable H3K27ac signal at the canonical promoter region of the *Robo3* gene region in pG-2 cells (Fig. S1D) [17]. Additionally, the repressive histone mark H3K27me3 occupied the gene body region spanning from exon 1 to 17, indicative of active repression of this area via Polycomb Repressive Complex 2 (PRC2) activity (Fig. S1D). In contrast, the region encoding *Robo3s* was negative for H3K27me3 and showed a strong accumulation of H3K27ac upon CAF treatment. Interestingly, we found that *Robo3s* expression was not restricted to the WAP-T mouse model, as the murine MMTV-Myc mammary carcinomas also showed specific expression of *Robo3s* together with a similar epigenetic profile at its gene locus and a promoter region-specific H3K4me3 peak in the proximity of exon 17 (Fig. S1E). Taken together, our data strongly support the presence of an alternative promoter region located between exon 17 and 23 that drives *Robo3s* expression independently of the full-length transcript canonical promoter.

The *Robo3s* transcript presents a potential open reading frame (ORF) spanning from exons 23 to 28, sharing the same stop-codon with long *Robo3* isoforms, that could give rise to a ROBO3s protein lacking the transmembrane domain encoded by exon 17 (Fig. 1F). We successfully verified the appearance of a ROBO3s band at the expected size of 28 kDa upon chemotherapy treatment in pG-2 and H8N8 cells (Fig. S1F, G). We could not identify any bands in the size range of full-length ROBO3 (150 to 200 kDa), that were specifically upregulated upon chemotherapy. To confirm the specificity of the putative ROBO3s band, we simultaneously treated pG-2 cells with CAF and *Robo3* siRNA and observed the disappearance of the ROBO3s band at 28 kDa (Fig. S1H, I). We then further confirmed the existence of the ROBO3s gene product by mass spectrometry (MS). We identified 6 peptides corresponding to a 40 % coverage of the predicted ROBO3s protein sequence in pG-2 cells upon CAF treatment. In all analyzed samples no peptide matching the N-terminal domain of the full-length protein could be detected (Figs. 1F, G and S2).

Based on our previous observations, pG-2 cells surviving CAF chemotherapy treatment showed increased aggressiveness [16–18]. Therefore, we questioned if *Robo3s* induction affects the tumor cell phenotype. First, we ensured that the treatment with siRNA targeting *Robo3* did not influence the proliferation of *Robo3s* negative cells (pG-2 neither express full-length *Robo3* nor *Robo3s* under basal growth conditions). (Fig. S1J). Interestingly, we observed that challenging pG-2 cells by reducing the concentration of fetal bovine serum (FBS) induced *Robo3s* expression (Fig. S1K). As FBS gradients are commonly utilized as a chemoattractant in Boyden chamber-based migration assays, we wondered if siRobo3 treatment could influence the motility of the tumor cells. Indeed, loss of Robo3s strongly reduced the capacity of pG-2 cells to migrate in vitro (Fig. 1H). Sphere formation assay under low serum concentrations additionally showed that loss of *Robo3s* impairs CSC of pG-2 cells (Fig. S1L). Collectively, we identified a new short isoform of the murine *Robo3* gene giving rise to an approximatively 28 kDa protein and that likely plays a role in increased tumor cell aggressiveness upon chemotherapy.

### Human BCs express a short ROBO3 variant that supports cell proliferation and migration in vitro

**Fig. 2:**
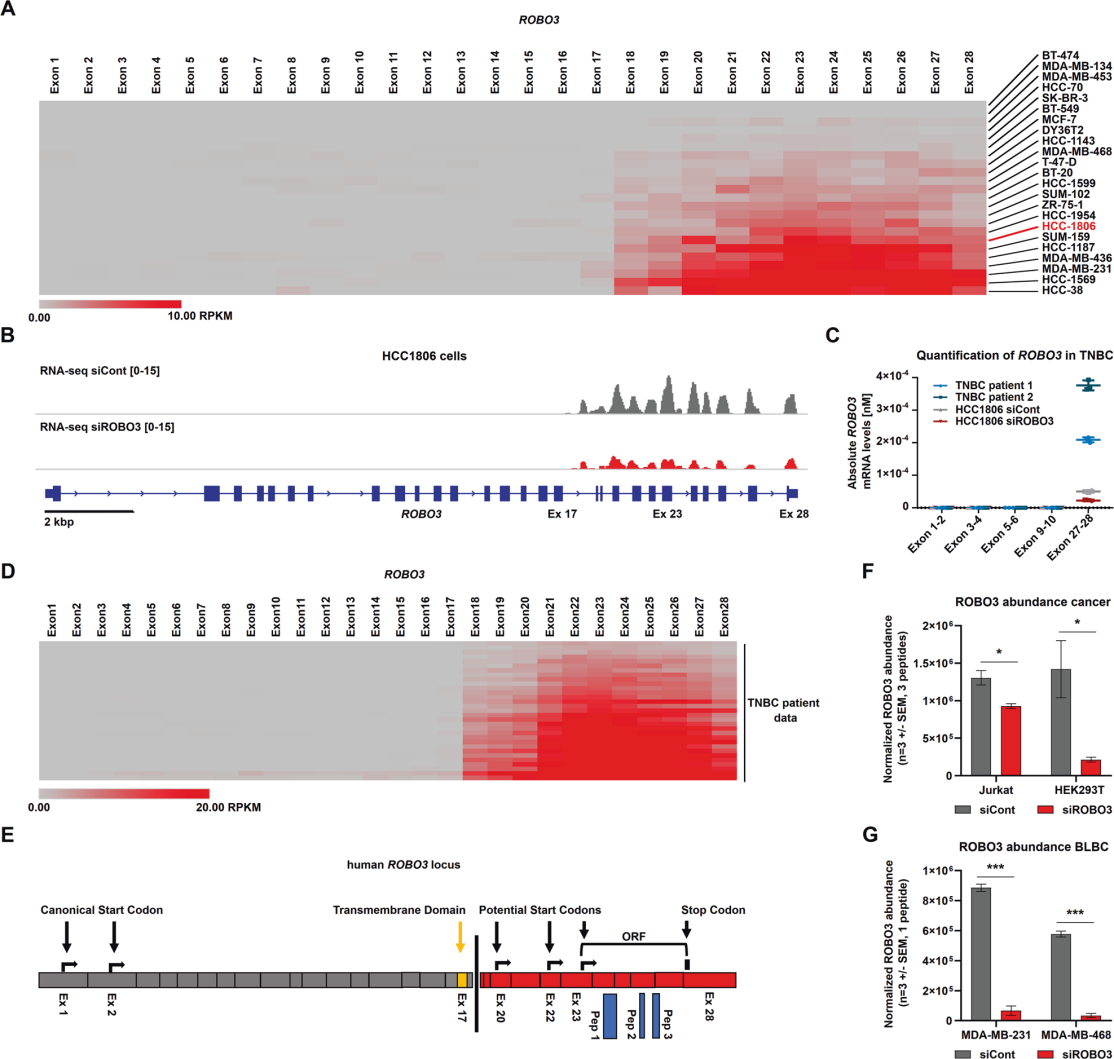
Human TNBC cells express a short ROBO3 variant that supports cell proliferation and migration in vitro. **A** Heatmap of *ROBO3* expression levels for each exon across 23 human BC cell lines derived from publicly available mRNA-seq data. Cell lines are sorted by the expression level of exon 23 based on reads per kilobase per million mapped reads (RPKM). **B** Reads coverage (mRNA-seq) of *ROBO3* in HCC1806 cells 72 h after treatment with control or anti-ROBO3 siRNAs (siCont and siROBO3, respectively). *ROBO3* signal was only detected in a region spanning from exon 17 to exon 28. siROBO3 treatment (red track) efficiently reduced the *ROBO3* signal compared to siCont (grey track). **C** qRT-PCRs with primer pairs raised against Exons 1-2, Exons 3-4, Exons 5-6, Exons 9-10, and Exons 27-28 of human *ROBO3*. Absolute mRNA levels were measured in two primary TNBC cultures (passage < p5) and in HCC1806 treated with siCont or siROBO3. A signal was only obtained for the primer pair Exons 27-28. **D** Heatmap of *ROBO3* expression levels (RPKM) for each exon across publicly available RNA-seq data sets of TBNC patients. **E** Schematic depiction of potentially translated (red) and non-translated (grey) *ROBO3* exons. ROBO3 peptide sequences identified by mass spectrometry are mapped against the amino acid sequence (blue). ORF analysis shows three possible start codons for translation. Canonical and newly predicted ORFs of ROBO3 terminate on an identical stop codon. The coding sequence for the ROBO3 transmembrane region is absent from the novel isoform (yellow). **F**, **G** Protein abundance of human ROBO3 quantified in Jurkat and HEK293T **F** as well as MDA-MB-231 and MDA-MB-468 cells **G** after ROBO3 siRNA knockdown in comparison to a siRNA control. The area under the curve of extracted fragment ion chromatograms was summed up and averaged for three peptides unique for ROBO3. **F**, **G:** Error bars represent mean ± SEM of three biological replicates, t-test: *p < 0.05; ***p < 0.001.

To determine if *Robo3s* expression could also be detected in human BC, we analyzed publicly available mRNA-seq datasets of BC cell lines. We observed that approximately 50% of the analyzed cell lines markedly express a short form of *ROBO3* (*ROBO3s* in this manuscript), slightly longer than the murine counterpart spanning from exon 18 to 28 under standard growth conditions (Fig. 2A). We selected the HCC1806 cell line to model BLBC in vitro as these cells harbor a medium *ROBO3s* expression level (Fig. 2A, S3A). mRNA-seq analysis of HCC1806 cells under normal growth conditions confirmed the presence of the previously identified *ROBO3s* transcript (Fig. 2B). Similar to the murine cell lines, investigation of epigenetic modifications in HCC1806 publicly available ChIP-seq datasets revealed the presence of an active alternative promoter located between exons 16 and 18 and characterized by strong RNA polymerase II (RNA Pol II), H3K4me3, and H3K27ac occupancy (Fig. S3B). Moreover, like in the mouse, the first 15 exons of the full-length gene were actively repressed via H3K27me3 occupancy. In line, RT-qPCR on two primary TNBC cells samples, as well as in HCC1806, confirmed the sole presence of the *ROBO3s* transcript (Fig. 2C).

We next leveraged publicly available human RNAseq datasets to characterize expression patterns of *ROBO3s* in normal and cancerous tissues. Primary BC lesions showed high *ROBO3s* expression levels in more than 50% of the cases in TNBC, BLBC and normal-like subtypes, and a slightly lower frequency in the other BC subtypes (Fig. 2D and Fig. S3C). Hinting toward a role of ROBO3s upregulation in cancer in humans, analyses of healthy and cancerous human tissues revealed that normal cells generally express lower *ROBO3s* levels (Fig. S3D, E). Among all analyzed normal human tissues, only the brain cortex showed a robust expression of the canonical long *ROBO3* transcript (GTEx dataset), further demonstrating that the exclusive *ROBO3s* detection in cancerous tissues is not resulting from sequencing artifacts (Fig. S3F). Interestingly, the expression pattern of normal tissues and their malignancies were conserved: for instance, liver and hepatocellular carcinomas almost completely lack ROBO3s expression, whereas breast and lung, and their respective cancers, showed the highest expression levels (Fig. S3D, E). Concluding, *ROBO3s* is the prevalent *ROBO3* transcript variant expressed in normal and cancerous tissues.

Prediction of potential open reading frames (ORF) in the human *ROBO3s* transcript revealed three possible translation start sites, all in frame with the full-length variant, leading to protein termination at the canonical ROBO3 stop-codon (Fig. 2E). Notably, one predicted ORF starting at exon 23 presented very high homology with murine ROBO3s (Fig. S4A). Unfortunately, most commercially available anti-human ROBO3 antibodies are raised against epitopes of the N-terminal extracellular domain, rendering the detection of ROBO3s impossible. Therefore, despite the assessment of different antibodies, western blot analyses failed to detect ROBO3s at the expected size range (between 18 and 50 kDa, data not shown). Hence, to elucidate whether ROBO3s is translated into protein in human cancer cells, we again took advantage of MS analysis. First, as *ROBO3s* expression is particularly high in HEK293T and Jurkat cells, we performed a ROBO3s peptides discovery approach in these cell lines. MS analysis identified three peptides corresponding to ROBO3s (Fig. 2F and S5). We next assessed the abundance of the identified ROBO3s peptide in the BLBC cell lines MDA-MB-231 and MDA-MB-468 (Fig. 2G and S4B, C). Strikingly, similar ROBO3s peptides were identified, and their levels were reduced by siRNA treatment. Here, again, no peptide matching the N-terminal domains of the full-length protein was detected.

**Fig. 3:**
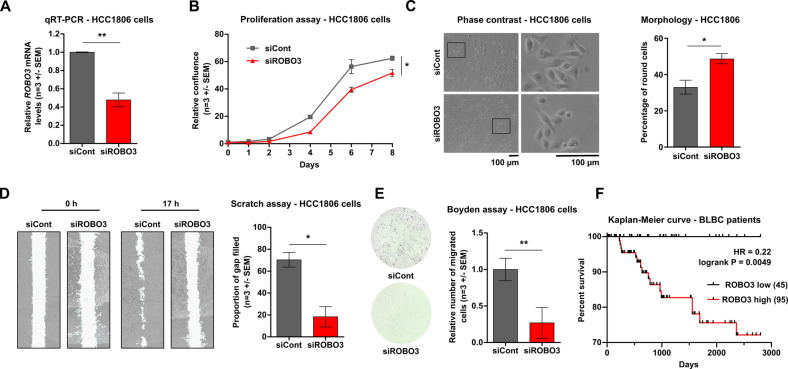
Human ROBO3s is essential for cell proliferation and cell migration. **A** Validation of siROBO3 knockdown in HCC1806 cells relative to siRNA control by qRT-PCR. **B** Proliferation assay of HCC1806 treated with siROBO3. Relative confluency was measured by Celigo® and normalized to day 0. Statistics were performed on the area under the curve (AUC). **C** Phase-contrast images of HCC1806 cells treated with siROBO3 or siRNA control (left panel). Quantification of the percentage of round cells (right panel). **D** Scratch assay gaps 0 h and 17 h after seeding. HCC1806 cells were treated with siROBO3 and siRNA control (siCont) respectively (left panel). Quantification of migratory cells based on relative filled gap area (right panel). **E** Boyden chamber based migration assay of HCC1806 cells treated with siROBO3 or siCont, respectively, and stained with crystal violet (left panel). A quantification of the migrated cell number is provided in the right panel. **F** Kaplan-Meier curve depicting the survival of BLBC patients (source TCGA-BRCA dataset) grouped according to *ROBO3* expression levels (*ROBO3* low *n* = 45 patients, *ROBO3* high *n* = 95 patients). Log-rank (Mantel-Cox) Test (*p* = 0.0049). Error bars represent mean ± SEM. All experiments were performed in three biological replicates. Unpaired t-test. *p < 0.05; **p < 0.01; ***p < 0.001.

Next, we investigated if ROBO3s has a tumorigenic function in BLBC cell lines. Knockdown of *ROBO3s* utilizing two different siRNAs targeting the short transcript significantly impaired HCC1806 cell proliferation (Fig. 3A, B, S4D, E). ROBO3s silencing also affected other BLBC cell lines in a *ROBO3s*-expression dependent manner: high *ROBO3s-*expressing MDA-MB-468 cells showed a marked proliferation reduction, while low *ROBO3s*-expressing HCC-70 cells were not significantly affected (Fig. S4F, G). Additionally, *ROBO3s* knockdown dramatically altered the morphology of HCC1806 cells, inducing a switch from an elongated motile shape to a rounder phenotype typical for less motile cells (Fig. 3C). Scratch and Boyden chamber assays further confirmed that *ROBO3s* loss heavily impaired HCC1806 cells motility (Fig. 3D, E). In line with these findings, an analysis of the TCGA-BRCA datasets showed that high *ROBO3* expression (ROBO3s^high^, as this was the only detected isoform, see Fig. S3C) strongly correlates with poor survival outcomes for BLBC patients (Fig. 3F). Together, our data revealed conservation of the ROBO3s isoform from mice to humans and demonstrated that it contributes to the oncogenic properties of human BLBC.

### Loss of ROBO3s impairs actin cytoskeleton structures and increases apoptosis

**Fig. 4:**
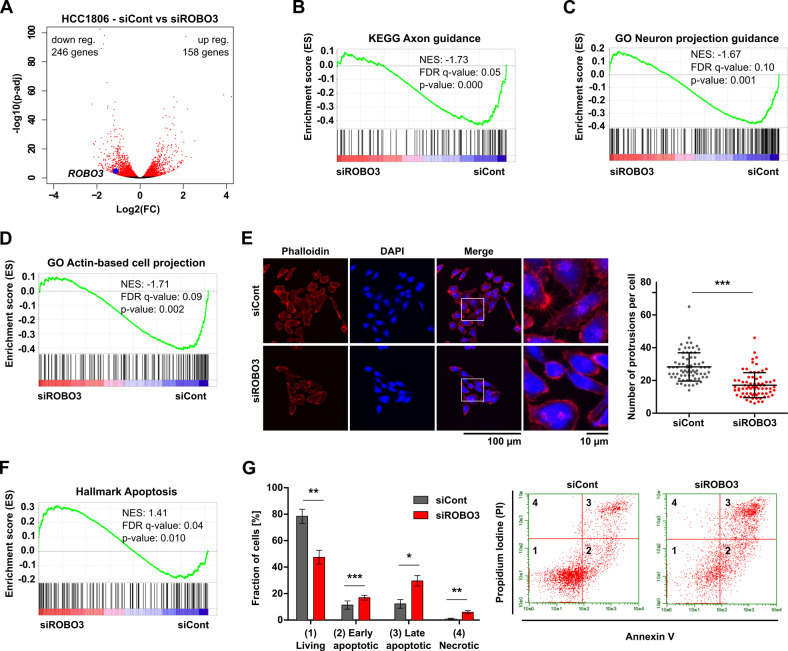
Loss of ROBO3s impairs actin cytoskeleton structures and increases apoptosis. **A** Differentially regulated genes in siROBO3 treated HCC1806 cells relative to siRNA control. 158 upregulated and 246 downregulated genes were identified (log2FC ≥ │1│, p-adj < 0.05). Genes exhibiting a significant regulation with a p-adj < 0.05 are highlighted in red. **B**–**D** Control HCC1806 cells enrich the “KEGG Axon guidance” **B**, the “GO Neuron projection guidance” **C** and the “GO Actin-based cell projection” **D** gene sets when compared to siROBO3 treated cell in Gene Set Enrichment Analysis (GSEA) (mRNA-seq). **E** Representative pictures of Phalloidin staining for the actin cytoskeleton in siRNA control and siROBO3 treated HCC1806 cells (left panel). Quantification of the number of actin-mediated cell protrusions in the respective conditions (right panel). **F** GSEA of the mRNA-seq data significantly enriched for the “Hallmark Apoptosis” gene set in the *ROBO3* knockdown condition. **G** Annexin V assay by Fluorescence Activated Cell Sorting (FACS) for HCC1806 treated with siROBO3 or siRNA control, respectively. 1: living cells. 2: early apoptotic. 3: late apoptotic. 4: necrotic. Quantification of cell populations (left panel). Distribution of cell populations and gating (right panel). Error bars represent mean ± SEM of three biological replicates, unpaired t-test. *p < 0.05; **p < 0.01; ***p < 0.001.

To understand the molecular mechanisms underlying ROBO3s-mediated cancer cell aggressiveness, we performed mRNA-seq in HCC1806 cells upon *ROBO3s* knockdown. Differential gene expression analysis identified 246 downregulated and 158 upregulated genes in siROBO3 treated cells (Fig. 4A). Gene set enrichment analysis (GSEA) identified a reduction of signatures specific for canonical ROBO3 function in neurons, suggesting that ROBO3s could share some functions with the long isoform (Fig. 4B, C). Additionally, the GSEA results suggested that ROBO3s loss in HCC1806 cells impairs actin regulatory pathways (Fig. 4D). To investigate this further, we performed Phalloidin stainings of actin fibers in HCC1806 cells after *ROBO3* knockdown and observed a significant reduction of actin-mediated cell protrusions, offering a possible mechanistic insight into the previously observed tumor cell migration impairments (Figs. 3D, E and 4E). Similarly, *ROBO3*^*high*^ BLBC tumors (TCGA-BRCA dataset) enriched for gene sets associated with positive regulation of axon extension and actin cytoskeleton regulation (Fig. S6A). In addition to the altered cytoskeletal dynamics, GSEA revealed that HCC1806 cells treated with siROBO3s were significantly enriched for apoptosis specific gene signatures (Fig. 4F). To confirm this finding, we performed Annexin V staining and observed a strong induction of programmed cell death in HCC1806 cells upon siROBO3s treatment (Fig. 4G). Together, these findings identify the induction of actin-mediated cell protrusions and inhibition of apoptosis as two mechanisms through which ROBO3 contributes to tumor aggressiveness.

### ROBO3s loss impairs the Hippo pathway and sensitizes BLBC cells to chemotherapy

**Fig. 5:**
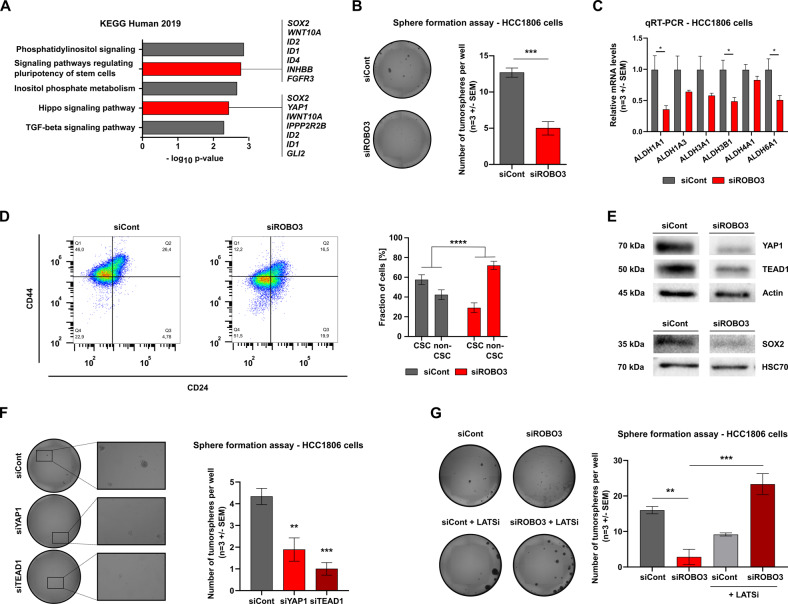
ROBO3s loss impairs the Hippo pathway and CSC characteristics. **A** Pathway enrichment analysis (EnrichR web tool) showing that genes significantly downregulated upon ROBO3 knockdown are enriched for the KEGG 2019 signature “Signaling pathways regulating pluripotency of stem cells” and “Hippo signaling pathway”. **B** Tumorsphere formation assay of control and siROBO3 treated HCC1806 cells (left panel). Quantification of tumorspheres number normalized to the control condition (right panel). **C** qRT-PCR showing a decrease of stem cell specific ALDH genes expression in siROBO3 treated HCC1806 cells. **D** Measurement of CD24 and CD44 positive population by fluorescence activated flow cytometry in HCC1806 cells treated with siCont or siROBO3. **E** Western blot of HCC1806 cells showing protein levels of SOX2, YAP1 and TEAD1 upon ROBO3 knockdown. **F** Tumorsphere formation assay of siControl, siYAP1 or siTEAD1 treated HCC1806 cells (left panel). Quantification of tumorspheres number normalized to the control condition (right panel). **G** Tumorsphere formation assay of siCont and siROBO3 treated HCC1806 cells, without or with LATSi (10 μΜ, left panel). Quantification of tumorspheres number normalized to the control conditions (right panel). **B**, **D**, **E**, **F** and **G**: Error bars represent mean ± SEM of three biological replicates, unpaired t-test or chi2-test **G**. *p < 0.05; **p < 0.01; ***p < 0.001.

To uncover the underlying molecular mechanisms, we utilized the Enrichr pathway enrichment tool (http://amp.pharm.mssm.edu/Enrichr/) and observed that many genes downregulated in HCC1806 cells upon siROBO3s treatment were associated with pathways regulating the pluripotency of stem cells (Fig. 5A). To test if *ROBO3s* is involved in HCC1806 stemness, we performed tumorsphere and colony formation assays upon ROBO3 knockdown. Strikingly, the number and size of spheres and colonies was drastically reduced upon *ROBO3s* loss (Fig. 5B and S6B, C). Furthermore, ROBO3s silencing led to a pronounced reduction of the *ALDH* family members (Fig. 5C) and of the CD44^high^/CD24^low^ cell population (Fig. 5D), both features associated with CSC-phenotypes [32]. Further supporting this finding, *ROBO3*^high^ BLBC lesions of the TCGA-BRCA dataset displayed a significant enrichment of mammary stem cell and EMT signatures in GSEA (Fig. S6D, E). In addition, the Hippo pathway, a critical pathway for CSC biology and self-renewal, was impaired upon ROBO3s knockdown, providing an interesting candidate for understanding the mechanism through which ROBO3s drives a CSC phenotype [33] (Figs. 5A and S6F). The YAP1 transcription factor (TF), which plays a pivotal role in Hippo signaling, was strongly downregulated upon ROBO3s loss (Figs. 5E and S6F) [34]. The level of TEAD1, one of the major YAP1 co-TFs, was also strongly reduced in siROBO3s treated cells (Fig. 5E). We therefore hypothesized that ROBO3s may support CSC properties by inducing the YAP1 transcriptional program. In support of this, the stem cell TF SOX2, known to be induced by YAP1, was also strongly downregulated in siROBO3 treated HCC1806 cells at the protein level (Fig. 5E) [35]. Silencing of both YAP1 and TEAD1 phenocopied the impairment of tumorsphere formation observed earlier upon ROBO3 knockdown (Figs. 5F and S6G, H). Noticeably, TEAD1 knockdown strongly accentuated the proliferation deficiency of siROBO3 treated cancer cells, pointing to sensitization of these cells to further interferences with the YAP1-signaling (Fig. S6I). YAP1 is regulated through the LATS1/2-kinases that catalyze its phosphorylation at serine 127. Consequently, YAP1 nuclear translocation is repressed and the protein is targeted for proteasome degradation [33]. Strikingly, the stimulation of YAP1 activity by using the LATS1/2 inhibitor TRULI (LATSi) completely restored the tumorsphere-forming capability of HCC1806 cells (Fig. 5G), further implicating YAP1 activity loss in the phenotype of ROBO3-silenced cells. Collectively, our data support a strong involvement of the ROBO3/YAP1-axis in promoting CSC features of BLBC cells.

**Fig. 6:**
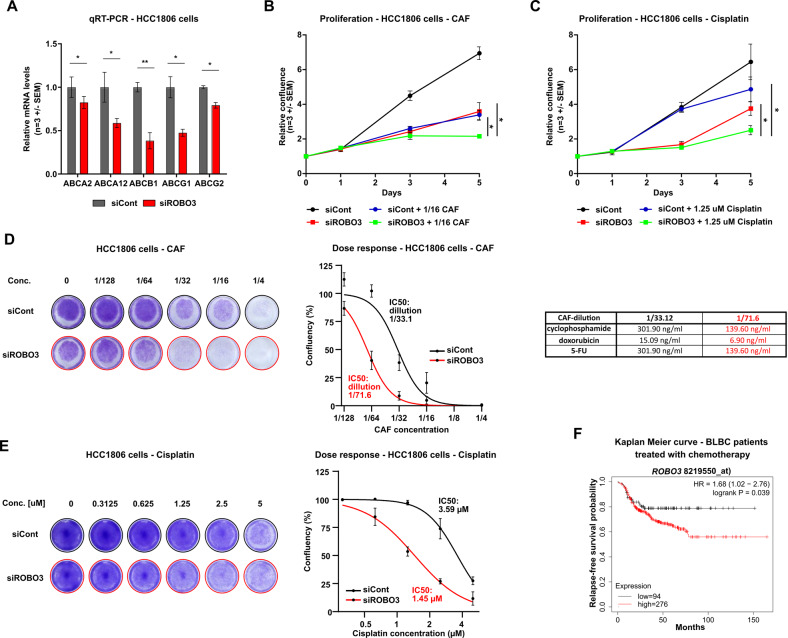
ROBO3s loss sensitizes BLBC cells to chemotherapy. **A** qRT-PCR of several ABC transporter genes upon ROBO3 knockdown or siRNA control treatment of HCC1806. **B** Proliferation assay of HCC1806 cells, treated with siROBO3, 1:16 CAF or in combination. **C** Proliferation assay of HCC1806 cells, treated with siROBO3, 1.25 μΜ cisplatin or a combination. Relative confluency was measured by Celigo® and normalized to day 0. Statistical analyses were performed on the AUC. **D**, **E** Dose response assay of siCont or siROBO3 treated HCC1806 cells and increasing doses of CAF or cisplatin. **F** Patient survival analysis for ROBO3^low^ and ROBO3^high^-expressing BLBC patients treated with chemotherapy (source: kmplot.com). **A**, **B**, **C**, **D** and **E**: Error bars represent mean ± SEM of three biological replicates, unpaired t-test. *p < 0.05; **p < 0.01; ***p < 0.001.

Since we originally identified *Robo3s* induction in murine tumor cells surviving chemotherapy, and since Hippo signaling has been connected to drug resistance [36], we hypothesized that ROBO3s expression could support tumor cell resistance by stimulating YAP1 activity. Notably, YAP1 was shown to promote the expression of several ABC-transporters and, thereby, support detoxification processes [37, 38]. Indeed, ROBO3s silencing resulted in significantly impaired expression of multiple ABC transporters involved in the resistance of cancer cells to numerous chemotherapeutic treatments (Fig. 6A) [39]. Based on these observations, we posit that inhibiting *ROBO3s* expression sensitizes BLBC cells to chemotherapy. To test this hypothesis, we assessed the resistance capacity of HCC1806 cells to CAF and cisplatin treatment upon *ROBO3* knockdown. As expected, combination of siROBO3 and chemotherapy (CAF or cisplatin, respectively) treatment significantly reduced the proliferation capacity of the cells compared to the single treatments (Fig. 6C, D and S6J–M). In line, ROBO3s knockdown strongly sensitized HCC1806 cells to increasing doses of CAF or cisplatin, pointing to a critical role of ROBO3s in rendering BLBC cells tolerant to cytotoxic therapies (Fig. 6D, E). Accordingly, *ROBO3*^low^ BLBC patients showed significantly better survival rates after adjuvant chemotherapy than *ROBO3*^high^ patients (Fig. 6F). Collectively, our results demonstrate that *ROBO3s* expression increases the CSC properties and drug tolerance of BLBC cells by activating the YAP1 signaling. Therefore, interfering with *ROBO3s* levels may represent an attractive strategy to sensitize BLBC to conventional chemotherapies, offering an opportunity for future targeted therapy approaches.

## Discussion

We identified *ROBO3* as a gene consistently upregulated upon chemotherapy survival in the basal-like mammary carcinoma WAP-T mouse model. Studies investigating the role of the ROBO family members have largely been limited to developmental biology with a particular focus on neurodevelopmental biology [40]. Despite increasing evidence implicating the SLIT/ROBO-signaling in oncologic pathways, the complexity and heterogeneity of ROBO proteins function remain insufficiently understood [41]. Notably, very recent works have pointed to the important role of *ROBO1* expression in driving oncogenic properties and cisplatin resistance in bladder and non-small cell lung cancer, respectively [42, 43]. In contrast, Chen and colleagues demonstrated a tumor-suppressive role of ROBO1 in inhibiting the proliferation of pancreatic cancer via the YY1-ROBO1-CCNA2-CDK2 axis [44]. Similarly, ROBO2 was shown to act as a tumor suppressor in pancreatic cancer, while being simultaneously a marker of poor prognosis in inflammatory BC [45, 46]. Together, these findings highlight how ROBO proteins are increasingly and recurrently being linked to cancer, however, there is a great need to further clarify these disparities and investigate the molecular mechanisms underlying them.

Studies assessing the function of ROBO3 in cancer are very scarce. In 2015, Han et al. showed that ROBO3 promotes pancreatic cancer growth and metastasis [31]. In contrast, Nakamura and colleagues recently reported a reduction of ROBO3 levels in invasive malignancies of the breast when compared with normal tissues and postulated a negative regulation of metastatic behaviors by Neural EGFL Like 2 (NELL2)/ROBO3-signaling [47]. By identifying a short, previously uncharacterized isoform of *ROBO3* (*ROBO3s*), our present study significantly contributes to a better understanding of the ROBO3-signaling complexity. We have demonstrated that, in mammary carcinomas, ROBO3s stimulates pathways involved in CSC maintenance, chemoresistance, and cell migration. ROBO3s lacks extracellular and transmembrane domains and, therefore, cannot bind extracellular ligands like NELL2 or SLIT, providing a possible explanation for the discrepancies with the study of Nakamura et al. The *ROBO3s* transcript spans from exons 18 to 28 in human cell lines and exons 23 to 28 in murine cells, respectively. Despite various studies on ROBO3 expression in neuronal systems and cancer and reports on several slightly divergent gene variants, this greatly shortened isoform of ROBO3 has, to our knowledge, never been described and uncovers a clear gap in our understanding of ROBO3 function [48–50]. Additionally, no specific intracellular signaling cascade has been identified downstream of ROBO3 [23]. This anonymity may explain why ROBO3 has been largely overlooked in studies of ROBO/SLIT signaling in cancer, and its discovery, therefore, opens the field to future investigations. A better comprehension of the ROBO3s-dependent signaling could establish this factor as an important prognostic factor or even as a new target for personalized therapies. Exact mechanistic insight into the intracellular signaling cascade of ROBO3 surpasses the scope of this study but uncovers an urgent demand for further research.

Analyses on a range of publicly available mRNA-sequencing datasets revealed that most normal mammary tissues, their respective malignancies and cell lines expressed *ROBO3s*, making it the most broadly expressed ROBO3 isoform. Interestingly, a short screen in other normal tissues and cancer entities (non-small cell lung cancers, prostate cancers, pancreatic cancers and liver cancers) confirmed the sole expression of the *ROBO3s* variant, suggesting that the importance of ROBO3s in cancer is likely not limited to BC.

ROBO3s was found upregulated in cancer cells surviving conventional chemotherapy. Indeed, we demonstrated a clear contribution of ROBO3s to cancer cell proliferation, migration, and stem cell characteristics. Stimulation of stem cell transcriptional program confers tumor cells’ resistance to various therapies by increasing detoxifying enzyme activity, immune tolerance and resistance to programmed cell death [51–54]. While the function of ROBO3 in stemness has not been investigated, research on the other family members ROBO1 and ROBO2 has stressed the involvement of the protein family in progenitor cell identity. Furthermore, ROBO1 and 2 were shown to control pancreatic progenitor identity by regulating YAP1 signaling [25]. Consistent with these findings, our data suggest that ROBO3s regulates stemness through Hippo-signaling. Upon ROBO3s knockdown, BLBC cells show impaired levels of Hippo pathway members YAP1 and TEAD1. Consequently, these cells reduce important stem cell associated features like the capability to form tumorspheres, high SOX2- and ALDH-genes expression and prominent CD44^high^/CD24^low^ cell population. Consequently, expression levels of several ATP-binding cassette transporters (ABC transporters) are reduced, explaining at least partially the gain of sensitivity to cytotoxic drugs. In line with our in vitro observations, high expression of *ROBO3s* in BLBC patients was associated with stem cell signatures and poor survival upon adjuvant chemotherapy, demonstrating the translational relevance of our in vitro functional studies. Interestingly, sustained YAP1/TEAD signaling is not only responsible for enhanced stem cell properties, but also strongly stimulates EMT transcriptional programs, cytoskeleton dynamics, and therefore correlates with poor patient survival [55–58]. Both axon guidance and tumor cell motility heavily leverage the actin cytoskeleton [59, 60]. It is therefore attractive to hypothesize that cancer cells hijack ROBO3s function to promote metastatic outgrowth.

In summary, this study identified *ROBO3s* as a factor associated with a multitude of aggressive tumor characteristics with potential prognostic value for tumor relapse and chemotherapy resistance.

## Supplementary information

<b>Authorship Statements</b>

<b>Reproducibility Checklist / Reporting Summary</>

<b>Werner_et_al_Supplements</b>

<b>Full and uncropped western blots</b>

<b>Figure S1</b>

<b>Figure S2</b>

<b>Figure S3</b>

<b>Figure S4</b>

<b>Figure S5</b>

<b>Figure S6</b>

## Data Availability

High throughput sequencing datasets generated and analyzed during the current study are available in the ArrayExpress repository (https://www.ebi.ac.uk/arrayexpress/) under the accession numbers E-E-MTAB-11344, E-MTAB-9589, E-MTAB-10744. Publicly available data analyzed during the current study and their respective accession numbers are listed in Table S3.
