# Supplementary material for: ROBO3s: a novel ROBO3 short isoform promoting breast cancer aggressiveness
Source: Cell Death Dis. 2022 Sep 3;13(9):762. doi: 10.1038/s41419-022-05197-7 (PMC9440919; doi:10.1038/s41419-022-05197-7)
Supplement: Supplementary file 1 — Authorship Statements [file 41419_2022_5197_MOESM1_ESM.pdf]

**From:** Marcel Werner <marcel.werner24@gmail.com>
**Sent:** Montag, 11. Juli 2022 22:03
**To:** Wegwitz, Florian
**Subject:** Re: Robo3 Manuscript Revision - Werner et al.
**Attachments:** Werner_et_al_Manuscript_R1_marked_MW.docx; Werner_et_al_Supplements_R1_marked_MW.docx

Hi Flo,

Hereby I declare that I agree with my authorship on this manuscript and the listed co-authors.

I attached both files with corrections marked in red and tracked changes. Also put a few comments.

Best,
Marcel

**From:** anna dyas <anna.dyas@hotmail.co.uk>
**Sent:** Dienstag, 16. August 2022 11:43
**To:** Wegwitz, Florian
**Subject:** Re: CDDIS-22-0839RR Initial Quality Check

Dear Flo,

Sorry I didn’t realise I had not already done this!

Yes thank you I agree with my co-authorship and the current author list (where I am second of 3 co-first authors, correct?).

Best wishes,
Anna

**From:** Iwan Parfentev <iwan.parfentev@gmail.com>
**Sent:** Dienstag, 12. Juli 2022 23:57
**To:** Wegwitz, Florian
**Subject:** Fwd: Robo3 Manuscript Revision - Werner et al.
**Attachments:** Werner_et_al_Manuscript_R1_marked.docx; Werner_et_al_Supplements_R1_marked.docx; 220709_AllFigs_Optimized.pdf

Dear Florian,
I agree with being a co-author and with the authorship list for this resubmission. The changes made seem logical to me and frankly, I liked the previous version a lot, too, and did not understand most of the critique from the reviewers. But then again, I am not an expert in the cancer field. Good luck with this version!

Best regards,

Iwan

**From:** Schmidt, Geske Elisabeth
**Sent:** Montag, 11. Juli 2022 17:15
**To:** Wegwitz, Florian
**Subject:** AW: Robo3 Manuscript Revision - Werner et al.
**Attachments:** Werner_et_al_Manuscript_R1_marked_GS.docx

Hi Flo,

was für eine ganze Mühe!!
Ich habe das erste Worddokument schon einmal durch.
I agree with the co-authorship and the author list!

Liebe Grüße
Geske

**From:** Iga M <iga.mieczkowska92@gmail.com>

**Sent:** Dienstag, 16. August 2022 13:10

**To:** Wegwitz, Florian

**Subject:** Re: FW: CDDIS-22-0839RR Initial Quality Check

Dear Florian,

I agree for the coauthorship.

Bests

Iga

**From:** Müller-Kirschbaum, Lukas Christoph
**Sent:** Freitag, 15. Juli 2022 08:17
**To:** Wegwitz, Florian
**Subject:** RE: Robo3 Manuscript Revision - Werner et al.

Dear Florian,

Congratulations on the great manuscript. I am very happy to have contributed to this study and agree on the co-authorship and list of authors.

Best regards,
Lukas Müller-Kirschbaum

**From:** Müller, Claudia <claudia.mueller@izi.fraunhofer.de>
**Sent:** Montag, 11. Juli 2022 16:14
**To:** Wegwitz, Florian
**Subject:** AW: Robo3 Manuscript Revision - Werner et al.
**Attachments:** Werner_et_al_Manuscript_R1_marked_CM.docx

Hello Florian,

I have enclosed some small corrections.

I agree with the co-authorship and with the actual author list!

Many thanks for your work!

Best,
Claudia

**From:** Kalkhof, Stefan <Stefan.Kalkhof@hs-coburg.de>
**Sent:** Montag, 11. Juli 2022 16:38
**To:** Müller, Claudia; Wegwitz, Florian
**Subject:** Werner_et_al_Manuscript_R1_marked.docx
**Attachments:** Werner_et_al_Manuscript_R1_marked.docx

Lieber Florian,
vielen Dank für das tolle Manuskript sowie das Angebot uns als Coautoren aufzunehmen, auch wenn leider der Nachweis der isoformspezifischen Peptiden in der Reanalyse der TNBC-Proteomdaten noch nicht erfolgreich war. Wie besprochen würde ich hier gerne nochmals gezielte Analysen der TNBC-Biopsien nachziehen, was gerne demnächst nochmals abstimmen könnten.
Im Anhang findest Du die meine wenigen Anmerkungen.
Beste Grüße und maximale Erfolgswünsche für die Submission
Stefan

**From:** Oliver Reinhardt <oliverreinhardt@gmx.net>
**Sent:** Donnerstag, 14. Juli 2022 06:45
**To:** Wegwitz, Florian
**Subject:** Re: Robo3 Manuscript Revision - Werner et al.

Hi Flo,

Thanks for your patience.
I agree with the co-authorship and the authors list.

Best
Oli

**From:** Urlaub, Henning
**Sent:** Freitag, 15. Juli 2022 06:33
**To:** Wegwitz, Florian
**Subject:** RE: Robo3 Manuscript Revision - Werner et al.

Lieber Herr Wegwitz – bitte entschuldigen Sie.
Ich bestätige hiermit die Autorenliste und bin mit der Änderung der Autorenliste einverstanden.
Herzlicher Gruss Henning Urlaub


************************************************
Prof. Dr. Henning Urlaub
Max Planck Institute for Multidisciplinary Sciences
Bioanalytical Mass Spectrometry Group
Am Fassberg 11
D-37077 Goettingen, Germany
phone: +49-(0)551-201-1060 (Office)/-1500 (Lab)
mobile: +49-(0)160-93905339
email: henning.urlaub@mpinat.mpg.de
http://www.mpinat.mpg.de/urlaub
************************************************
University Medical Center Goettingen
Bioanalytics
Institute for Clinical Chemistry
Robert Koch Strasse 40
D-37075 Goettingen, Germany
phone: +49-(0)551-39-65192 (Office) /-12501 (Lab)
mobile: +49-(0)160-93905339
Email: hurlaub@gwdg.de
http://www.clinchem.med.uni-goettingen.de/content/forschung/30.html
*************************************************

**From:** Alves, Frauke
**Sent:** Mittwoch, 13. Juli 2022 17:30
**To:** Wegwitz, Florian
**Subject:** Robo3 Manuscript revision

Dear Florian,

herewith I state that I agree with my co-authorship and with the actual author list and revised manuscript.

ROBO3s: a novel ROBO3 short isoform promoting breast cancer aggressiveness

Marcel Werner[1,2*], Anna Dyas[1,3,4*], Iwan Parfentev[5*], Geske E. Schmidt[6], Iga K Mieczkowska[1], Lukas C Müller-Kirschbaum[1], Claudia Müller[7], Stefan Kalkhof[7], Oliver Reinhardt[8], Henning Urlaub[5,9], Frauke Alves[8,10], Julia Gallwas[11], Evangelos Prokakis[11#], Florian Wegwitz[11#]

mit freundlichen Grüßen
with best regards

*Frauke Alves*

------------------------------------------------------------
Prof. Dr. med. Frauke Alves

Translational Molecular Imaging

Institute of Diagnostic and Interventional Radiology
Clinic of Hematology and Medical Oncology
University Medical Center Goettingen (UMG)
Robert Koch Str. 40
37075 Göttingen
Tel: +49 (0)551 39 66991

&
Max Planck Institute for Multidisciplinary Sciences
City Campus
Hermann Rein Str. 3
37075 Göttingen
Tel: +49 (0)551 201-31 655
Fax: +49 (0)551 201-31 644

**From:** Gallwas, Julia <julia.gallwas@med.uni-goettingen.de>
**Sent:** Freitag, 15. Juli 2022 07:25
**To:** Wegwitz, Florian

Lieber Herr Wegwitz.,

vielen Dank für die tollte gemeinsame Zeit mit Ihnen und dem Team. Ich weiss sehr zu schätzen was Sie Leisten!

Im Bezug auf unsere aktuelle Arbeit möchte ich Ihnen noch die Zusage von meiner Seite geben.

I declare that I agree with my authorship on the manuscript “ROBO3s: a novel ROBO3 short isoform promoting breast cancer aggressiveness” and the modified list of authors.

Best regards
Julia Gallwas

Prof. Dr. med. Julia Gallwas
Ärztliche Direktorin
UNIVERSITÄTSMEDIZIN GÖTTINGEN
GEORG-AUGUST-UNIVERSITÄT
Klinik für Gynäkologie und Geburtshilfe
Robert-Koch-Straße 40
37075 Göttingen
Tel. Sekretariat: 0551/39-62501
Fax. Sekretariat: 0551/39-62192
Julia.Gallwas@med.uni-goettingen.de

**From:** Prokakis, Evangelos
**Sent:** Freitag, 12. August 2022 08:37
**To:** Wegwitz, Florian
**Subject:** Re[2]: FW: Decision letter for CDDIS-22-0839Accepted in Principle

Hello Florian,

I agree with the submission of the manuscript at Cell Death and Disease.

Best wishes,

Evangelos

--
Sent from Outlook Email App for Android

Δευτέρα, 08 Αυγούστου 2022, 02:20μ.μ. +02:00 from "Wegwitz, Florian" fwegwit@gwdg.de:
