# Supplementary material for: ROBO3s: a novel ROBO3 short isoform promoting breast cancer aggressiveness
Source: Cell Death Dis. 2022 Sep 3;13(9):762. doi: 10.1038/s41419-022-05197-7 (PMC9440919; doi:10.1038/s41419-022-05197-7)
Supplement: Supplementary file 3 — Werner_et_al_Supplements [file 41419_2022_5197_MOESM3_ESM.docx]

**Supplemental Data**

**ROBO3s: a novel ROBO3 short isoform promoting breast cancer aggressiveness**

**Marcel Werner^1,2*^, Anna Dyas^1,3,4*^, Iwan Parfentev^5*^, Geske E. Schmidt^6^, Iga K. Mieczkowska^1^, Lukas C. Müller-Kirschbaum^1^, Claudia Müller^7^, Stefan Kalkhof^7^, Oliver Reinhardt^8^, Henning Urlaub^5,9^, Frauke Alves^8,10^, Julia Gallwas^11^, Evangelos Prokakis^11#^, Florian Wegwitz^11#^**

1) Department of General, Visceral and Pediatric Surgery, University Medical Center Göttingen, Göttingen, Germany

2) Chromosome Dynamics and Genome Stability, Institute of Epigenetics and Stem Cells, Helmholtz Zentrum München, Munich, Germany

3) International Max-Planck Research School for Molecular Biology, Göttingen, Germany

4) Early Cancer Institute, University of Cambridge, Department of Oncology, Hutchison Research Centre, Box 197 Cambridge Biomedical Campus, Cambridge, CB2 0XZ

5) Bioanalytical Mass Spectrometry group, Max Planck Institute for Multidisciplinary Sciences, Göttingen, Germany

6) Department of Gastroenterology, Gastrointestinal Oncology and Endocrinology, University Medical Center Göttingen, Göttingen, Germany

7) Department of Therapy Validation, Fraunhofer Institute for Cell Therapy and Immunology, Leipzig, Germany

8) Translational Molecular Imaging, Max-Planck Institute for Multidisciplinary Sciences, Göttingen, Germany

9) Bioanalytics, Institute of Clinical Chemistry, University Medical Center Göttingen, Göttingen, Germany

10) Department of Hematology and Medical Oncology, University Medicine Goettingen, Göttingen, Germany

11) Department of Gynecology and Obstetrics, University Medical Center Göttingen, Göttingen, Germany

**Supplemental Methods**

**siRNA transfection**

Cells were reverse transfected with siRNA using RNAiMAX (Invitrogen) in OPTIMEM medium (Gibco) according to the manufacturer's recommendations. The medium was replaced by normal culture medium after 6-12 h. Cells were seeded for functional assays 72 h after siRNA transfection or subjected to extraction of proteins or RNA. All siRNAs used in this study are listed in Table S7.

**Analysis of growth kinetics**

For analysis of growth kinetics, cell lines were seeded in 24-well plates (5*10^3^ cells/well) in the respective culturing media. Confluency was measured every 48 hours using a Celigo® imaging cell cytometer (Nexcelom) for 5-8 days.

**Gap closure assay**

For gap closure assays 70 µL of a 5*10^5^ cell/mL suspension were seeded on either side of a gap closure insert (Ibidi) in a 24-well plate. 24 h later, cells were serum-starved for 1 h before the inserts were removed. Cells were washed with PBS and supplemented with full culturing medium. After 12 h growth time phase-contrast photographs were taken every 5 hours.

**Migration assay**

Boyden chambers were equilibrated with serum-free medium. Next, the equilibration medium was removed and 5*10^4^ cells in 250 µL serum-free medium were seeded in the Boyden chamber. Cells were incubated at 37 °C for 3 h. Boyden chambers were then transferred to a 24-well plate with full growth medium. 48 h later, chambers were washed with PBS and fixed in 500 µL 4 % PFA for 10 min. Boyden chambers were washed with PBS and cells remaining on the upper side of the Boyden chambers were removed. Cells that migrated to the lower face of the membrane were then stained with either crystal violet (0.1 % in 20 % EtOH) and photographed with a stereomicroscope (SteREO Lumar. V12, Zeiss).

**Annexin V assay**

Cells pelleted from cell culture supernatants were washed 2X with cold PBS. Adherent cells were collected by trypsinizing, washing 2X with cold PBS and centrifugation. The combined cell pellets were resuspended in 1X Binding Buffer and filtered. The cell suspensions were then diluted to 1*10^6^ cells/mL from which 100 µL were transferred to a sterile Eppendorf tube. Per 100 µL of cell suspension, 2.5 µL of Annexin-FITC (Southern Biotech, cat nr. 10040-02) and 1 µL of propidium Iodide (Sigma Aldrich, cat nr. [25535-16-4](https://www.sigmaaldrich.com/catalog/search?term=25535-16-4&interface=CAS%20No.&N=0&mode=partialmax&lang=de&region=DE&focus=product)) were added. Samples were gently mixed and incubated for 15 min at room temperature (RT) in the dark. Samples were diluted with 400 µL of 1X Binding Buffer and immediately analyzed by flow cytometry (Guava® easyCyte™, Millipore).

**Phalloidin staining**

For phalloidin staining, 5*10^4^ cells were seeded on coverslips in 6-well plates. 48 h later, cells were washed with PBS and fixed with 4 % PFA in PBS for 5 min. Coverslips were then washed 3 times for 3 min in PBS and stained with 50 µL of PBS with Phalloidin-Alexa 555 (1:400, Abnova) and DAPI (1:1,000) for 2 h in a humidified dark chamber. Coverslips were washed 3 times in PBS for 3 min and mounted onto glass slides in 25 µL Mowiol 4-88 (Fluka). After air-drying overnight and coverslips were imaged on a fluorescence microscope Axio Scope.A1 (Zeiss).

**Sphere and colony formation assay**

HCC1806 cells were trypsinized and the cell concentration was estimated using trypan blue to ensure an accurate count of living cells. For tumor spheres, cells were then seeded in a low-adherent 96-well plate at a concentration of 1000 cells per well in technical and biological triplicates. For colonies, 1000 cells per well were seeded in a 6-well plate in one technical replicate and biological triplicates. Every condition was tested in biological triplicates. The number, analyses on growing spheres and colonies were assessed using a Celigo® imaging cell cytometer and ImageJ, respectively.

**Chemotherapy resistance assay**

For treatment with 5-FU, HCC1806 cells were trypsinized and the cell concentration was estimated using trypan blue to ensure an accurate count of living cells. 4,000 cells in 200 µL normal culture medium per well were seeded in a 96-well plate. For treatment with Cyclophosphamide, 5-FU and Doxorubicin and Cisplatin (CAF), HCC1806 cells were reverse transfected in 24-well plates as previously described. After 24 hs, the media was replaced with chemotherapy for 48 hs. The different chemotherapy concentrations used in this study are listed in Table S2. Resistance to chemotherapy was assessed using Celigo® imaging cell cytometer measurements over a time course. The IC50 of CAF and cisplatin was calculated using the non-linear regression analysis with Graphpad Prism v.8.0.1.

**Protein extraction**

Cells were in 6-wells were washed with PBS and subsequently lysed with 300 µL ice-cold RIPA buffer supplemented with protease and phosphatase inhibitors (10 mM BGP, Sigma-Aldrich; 10mM NEM, Sigma-Aldrich; 10 mM Pefabloc, Roth; Aprotinin/Leupeptin, Roth; IAA, Sigma-Aldrich) on ice. Lysed cells were scraped and transferred to Eppendorf tubes. Samples were then sonicated for 10-20 min in a Bioruptor (Pico Sonication device, Diagenode) (30 s ON/OFF). After protein concentration measurement with the BCA method (Pierce), appropriate amounts of 6x Laemmli buffer was added to protein lysates, followed by a denaturation step of 5 min at 95° C.

**Western blotting**

Equal amounts of protein were separated by 12 % SDS-PAGE and then transferred to nitrocellulose or PVDF membranes. Membranes were washed in TBS-T and blocked in 5 % skimmed milk in TBS-T for 1 h at RT. Primary antibodies were added overnight at 4^o^C (0.05 % NaN_3_ in 5 % milk in TBS-T). After three wash steps (TBS-T, 5 min), secondary antibodies conjugated with horseradish peroxidase-conjugated were applied for 1 h at RT. Finally, membranes were washed (3 times TBS-T for 5 min) and developed in a ChemiDoc TM MP System (Bio-Rad Laboratories) using Immobilon Western Blot HRP Substrate (Millipore) Reagent. Primary and secondary antibodies are listed in Table S5 and Table S6.

**Gene expression analysis by qRT-PCR**

RNA isolation from cell cultures was carried out using QIAzol® (Qiagen) according to the manufacturer’s instructions. The quality and integrity of the RNA were evaluated by agarose gel electrophoresis. RNA was reverse transcribed into cDNA with a M-MuLV reverse transcriptase kit (New England Biolabs). For quantification of relative gene expression, cDNA amplification was detected by SYBR green using a CFX Biorad system (Bio-Rad Laboratories). The following QRT-PCR protocol was used: 95° C for 2 min, 40 cycles of 95° C for 10 s and 60° C for 30 s, followed by a melting curve between 67° C and 95° C of 0.5° C/s. PCR reactions for each sample were repeated in triplicates. The integrity of the amplified products was confirmed by melting curve analysis. *RPLP0* was used as endogenous control. Primers for qPCR were designed using the Primer3 tool (biotools.umassmed.edu/bioapps/primer3_www.cgi) with input sequences from the UCSC genome browser (https://genome.ucsc.edu/). Primers were tested for multiple binding sites via BLAT tool of UCSC genome browser. Primers used in this study are listed in Table S2.

**RNA library preparation**

RNA samples were prepared using the TruSeq RNA Library Prep Kit v2 (Illumina) according to the TruSeq RNA Sample Preparation v2 Guide (Illumina). Library concentration was estimated using a Qubit fluorometer (Invitrogen) and the quality of samples was validated on a high-sensitivity DNA Chip using a Bioanalyzer 2100 (Agilent). Barcoded samples were finally pooled to a concentration of 2 nM. Sequencing reactions were performed on a HiSeq4000 (Illumina), single-end, 50 bp read length, at the NIG (NGS Integrative Genomics Core Unit, Goettingen, Germany).

**mRNA sequencing analysis**

RNA sequencing raw data were processed in the GALAXY environment ^1^ provided by the GWDG (https://galaxy.gwdg.de/). FastQC was carried out to assess the quality of the FASTQ files. Data were trimmed using the FASTQ Trimmer tool (v1.1.1) with an offset of 11 from the 5’ end. Next, single reads were aligned to the reference genome GRCh38 (Hg38) using RNA STAR (v2.6.0b-2) and assigned to the respective genes with FeatureCounts (v1.6.0.2). DESeq2 (v2.11.40.1) was carried out to normalize gene expression values and estimate differential gene expression.

EnrichR (http://amp.pharm.mssm.edu/Enrichr/) and Gene Set Enrichment Analysis (GSEA, Broad Institute) were used to identify gene signatures enriched in the different experimental conditions. For these analyses, genes with a base mean expression lower than 15 normalized counts were considered absent. A threshold of p-value less than or equal to 0.05, and a Log2(fold change) greater than 0.5 or less than -0.5 was utilized in EnrichR analyses. GSEA (v4.1.0) was run with the parameters Max size 1000, Min size 15, Number of markers 1000, Collapse dataset ‘False’, Permutation “gene_set” and the remaining parameters were set to default ^2^. High throughput sequencing data produced in this study were deposited at the ArrayExpress platform (<https://www.ebi.ac.uk/arrayexpress/>) under the accession number E-MTAB-9589 and E-MTAB-10744, and are listed in Table S3.

**Analysis of publicly available mRNA-seq and ChIP-Seq datasets**

Publicly available RNA-seq and ChIP-Seq datasets stored at the EBI (European Bioinformatics Institute, https://www.ebi.ac.uk/) were imported into the Galaxy environment provided by the GWDG (list provided in Table S3). Processing of the raw mRNA-seq data and alignment to the hg38 or the mm10 (GRCm38) reference genomes was performed as described previously ^3^. Duplicates in ChIP-seq raw data were removed with RmDup v2.0.1) and reads were aligned to hg38 or mm10 using bowtie2 (v3.2.0.0.). To visualize the mRNA- and ChIP-seq tracks, bigwig files were generated with BamCoverage (Version 3.2.0.0.0) with the following settings: bin size 25, normalization method: reads per kilobase per million (RPKM), smooth length: 75 bases. Occupancy profiles were visualized using the Integrative Genomics Viewer (IGV v2.4.8).

Heatmap of *ROBO3*-Exon expression in BC-cell lines and patients: bigwig files computed in the previous step were utilized to generate a matrix of exon specific *ROBO3* expression with computeMatrix (v3.2.0.0.0) and following parameters: compute matrix mode ‘scale-regions’, ‘regionBodyLength’ = 24, ‘binSize’ = 24, ‘—sortRegions’ = keep and ‘saveMatrix’ ^4^. The webtool Morpheus (<https://software.broadinstitute.org/morpheus/>) was used to generate the heatmaps. The *ROBO3*-Exon region file is provided in Table S8.

Kaplan-Meier plots

**Sample preparation for Mass Spectrometry analysis**

Samples were incubated at 70 °C for 10 min and sonicated at 4 °C for 10 min (Bioruptor, Diagenode) in 30 s intervals. Cysteine residues were reduced and alkylated by the addition of 10 mM Tris-(2-carboxyethyl)-phosphin and 30 mM chloroacetamide and incubation at 1000 rpm for 45 min in the dark. Subsequently, proteins were extracted and digested according to the SP3 protocol ^5^. Briefly, paramagnetic beads coated with carboxylate functional groups (Thermo Fisher Scientifc) were added in 10x excess (w/w) of the protein content, followed by the addition of ethanol to a final concentration of 50% (v/v). Samples were incubated for 10 min at 1000 rpm for protein binding and beads were collected at the tube wall with a magnet. Beads were then washed three times with 80% ethanol (v/v) and resuspended in 100 mM triethylammonium bicarbonate buffer (Sigma-Aldrich). Proteins were enzymatically digested overnight at 1000 rpm and 37 °C after the addition 1:20 trypsin (w/w). Finally, beads were separated from the supernatant twice with a magnet and tryptic peptides were dried in a Speedvac concentrator.

For the discovery approach, peptides were fractionated by high pH reversed-phase chromatography using an Agilent 1100 Series HPLC system. Peptides were resuspended in 5% acetonitrile (ACN) (v/v) containing 10 mM NH_4_OH and loaded onto an Xbridge C18 column (Waters) at a flow rate of 60 µL/min. Following, peptides were separated along a gradient of 5% to 55% buffer B (80% ACN, 10 mM NH_4_OH, buffer A contained no ACN) over the course of 96 min. Fractions were collected every minute, pooled to overall 24 fractions and were dried prior to LC-MS analysis.

**LC-MS analysis**

Fractions obtained from high pH reversed-phase chromatography were submitted to liquid chromatography coupled to mass spectrometry (LC-MS) analysis on a Dionex UltiMate 3000 RSLCnano system, which was connected to a Q Exactive HF-X or an Orbitrap Exploris 480 mass spectrometer (all from Thermo Fisher Scientific). Peptides were resuspended in 2.5% ACN, 0.1% trifluoroacetic acid (TFA) (v/v) and injected onto a C18 PepMap100 µ-Precolumn (0.3 x 5 mm, 5 µm, Thermo Fisher Scientific). Subsequently, peptides were separated on an in-house packed main column (75 µm x 30 cm, Reprosil-Pur 120C18-AQ, 1.9 µm, Dr. Maisch GmbH) at 300 nl/min flow rate. The chromatographic gradient consisted of the following steps: 5% to 8% buffer B (80% ACN, 0.08% formic acid, v/v) from 0 to 5 min, 8% to 36% buffer B from 5 to 42 min and 36% to 45% buffer B from 42 to 45.9 min, followed by an equilibration sequence with 90% and 5% buffer B. The overall method duration was 58 min. Precursor spectra (MS1) were acquired with 120,000 resolution (full width at half maximum, FWHM), 1e6 automatic gain control (AGC) target, 50 ms maximum injection time, and a scan range from 300 to 1,600 m/z. The 40 most abundant precursor ions per duty cycle with a charge state between +2 and +4 were isolated individually with a 1.4 m/z isolation window and were fragmented with a normalized collision energy of 30. Fragment ion (MS2) spectra were acquired with 15,000 resolution (FWHM), 8e3 minimum AGC and 2e5 target AGC, and 54 ms maximum ion injection time. Selected precursor ions were excluded from further fragmentation events for 30 s. Technical replicate injections were performed with an inclusion list for all theoretical m/z values of tryptic peptides of C-terminal Robo3 (starting from exon 23) with a charge between +2 and +4 and within the m/z range of 300 to 1,600.

After the identification of murine and human Robo3 candidate peptides (see below), targeted acquisitions of non-fractionated samples were performed with the same gradient and an inclusion list for said peptides with a 10 minute window around the observed elution time. In each duty cycle, an MS1 scan followed by a parallel reaction monitoring (PRM) event was performed with 120,000 resolution (FWHM), 2e5 AGC target, 200 ms maximum ion injection time, and 0.8 m/z isolation window around the individual targeted masses. For Jurkat and HEK293 cells, PRMs were performed with 240,000 resolution (FWHM), 1e6 AGC target, and 3 s maximum ion injection time. For breast cancer cell lines, the AGC target was further raised to 5e6.

**Database search for the discovery approach**

Raw acquisition files were analysed with Maxquant version 1.6.2.10 with default parameters ^6^. Searches were performed against reviewed human and mouse reference proteome databases derived from Swiss-prot/UniProtKB (human: 20,362 sequences, retrieved on 05/24/20; mouse: 17,042 sequences, retrieved on 05/14/20). Several sequences of C-terminal Robo3 were appended to account for possible alternative open reading frames and translation start sites.

**Data analysis of targeted acquisition data**

PRM acquisition files were analysed with Skyline version 20.1 ^7^. Spectral libraries of Robo3 peptides were generated from Maxquant database search results. Extracted ion chromatograms from all fragment ions were transformed by Savitzky-Golay smoothing and were filtered for high signal intensity, high signal-to-noise ratio, and the absence of co-eluting ions. Retention times and peak boundaries were adjusted manually if necessary and peak areas under the curve were exported. Finally, Peak areas were normalized against the total ion chromatogram and a Robo3 abundance value was calculated as an average of all contributing peptide peak areas.

**References**

1 Afgan E, Baker D, van den Beek M, Blankenberg D, Bouvier D, Čech M *et al.* The Galaxy platform for accessible, reproducible and collaborative biomedical analyses: 2016 update. *Nucleic Acids Res* 2016; **44**: W3–W10.

2 Subramanian A, Tamayo P, Mootha VK, Mukherjee S, Ebert BL, Gillette MA *et al.* Gene set enrichment analysis: A knowledge-based approach for interpreting genome-wide expression profiles. *Proc Natl Acad Sci U S A* 2005; **102**: 15545–15550.

3 Mieczkowska IK, Pantelaiou-Prokaki G, Prokakis E, Schmidt GE, Müller-Kirschbaum LC, Werner M *et al.* Decreased PRC2 activity supports the survival of basal-like breast cancer cells to cytotoxic treatments. *Cell Death Dis* 2021; **12**: 1118.

4 Ramírez F, Ryan DP, Grüning B, Bhardwaj V, Kilpert F, Richter AS *et al.* deepTools2: a next generation web server for deep-sequencing data analysis. *Nucleic Acids Res* 2016; **44**: W160–W165.

5 Hughes CS, Moggridge S, Müller T, Sorensen PH, Morin GB, Krijgsveld J. Single-pot, solid-phase-enhanced sample preparation for proteomics experiments. *Nat Protoc* 2019; **14**: 68–85.

6 Cox J, Mann M. MaxQuant enables high peptide identification rates, individualized p.p.b.-range mass accuracies and proteome-wide protein quantification. *Nat Biotechnol 2008 2612* 2008; **26**: 1367–1372.

7 Pino LK, Searle BC, Bollinger JG, Nunn B, MacLean B, MacCoss MJ. The Skyline ecosystem: Informatics for quantitative mass spectrometry proteomics. *Mass Spectrom Rev* 2020; **39**: 229–244.

**Supplemental Tables**

**Table S1: cell lines used in this study**

| **Cell line** | **Specie** | **Tissue of Origin** | **Growth medium** |
| --- | --- | --- | --- |
| HCC1806 | Human | Mammary carcinoma | RPMI GlutaMAX  (Gibco, #61870-010) |
| HCC70 | Human | Mammary carcinoma |  |
| MDA-MB-231 | Human | Mammary carcinoma | DMEM high glucose GlutaMAX  (Gibco, #61965-026) |
| MDA-MB-468 | Human | Mammary carcinoma |  |
| Jurkat | Human | T cell leukemia |  |
| HEK293T | Human | Kidney |  |
| pG-2 cells | Mouse | Mammary carcinoma |  |
| H8N8 | Mouse | Mammary carcinoma |  |

**Table S2: concentrations of chemotherapies used in this study**

| **Chemotherapy** | **Cyclophosphamide** | **Doxorubicin** | **5-FU** | **Cisplatin** |
| --- | --- | --- | --- | --- |
| **CAF 1:4** | **2.5 µg/ml** | **125 ng/ml** | **2.5 µg/ml** | **-** |
| **CAF 1:16** | **625 ng/ml** | **31.25 ng/ml** | **625 ng/ml** | **-** |
| **CAF 1:32** | **312.5 ng/ml** | **15.6 ng/ml** | **312.5 ng/ml** | **-** |
| **CAF 1:64** | **156.25 ng/ml** | **7.8 ng/ml** | **156.25 ng/ml** | **-** |
| **5-FU** | **-** | **-** | **312.5 ng/ml** | **-** |
| **Cisplatin 625 nM** | **-** | **-** | **-** | **625 nM** |
| **Cisplatin 1.25 µM** | **-** | **-** | **-** | **1.25 µM** |
| **Cisplatin 5 µM** | **-** | **-** | **-** | **5 µM** |

**Table S3: mRNA- and ChIP-seq accession numbers used in this study**

| **Tissue** | **Dataset** | **Accession** |
| --- | --- | --- |
| pG-2 (*in vitro* CAF treatment) | RNA sequencing | E-MTAB-9547 |
|  | ChIP-sequencing | E-MTAB-9584 |
| H8N8 (*in vitro* CAF treatment) | RNA sequencing | E-MTAB-11344 |
| H8N8 (*in vivo* CAF treatment) | RNA sequencing | E-MTAB-9589 |
| HCC1806 | RNA sequencing | E-MTAB-10744 |
|  | H3K4me3 ChIP-sequencing | SRR7621260 |
|  | H3K27me3 ChIP-sequencing | SRR7621267 |
|  | H3K27Ac ChIP-sequencing | SRR7621268 |
|  | Pol II ChIP-sequencing | SRR7621266 |
| Mouse brain | RNA sequencing | SRR6261044 |
| MMTV-Myc cells | H3K4me3 ChIP-sequencing | SRR2104573 |
|  | H3K27me3 ChIP-sequencing | SRR2104574 |
|  | H3K27Ac ChIP-sequencing | SRR2104575 |
| Breast cancer cell lines | Breast cancer cell lines | PRJNA251383 |
| Breast cancer tumors | TNBC tumors | PRJNA251383 |
| Various human tissue | Various human tissues | PRJNA494560 |
| Various human cancers | Hepatocellular carcinoma | PRJNA648076 |
|  | Cervical squamous cell carcinoma | PRJNA705749 |
|  | Non-small-cell lung carcinoma | PRJNA520852 |
|  | Pancreatic ductal adenocarcinoma | PRJNA471423 |
|  | Ovarian carcinoma | PRJNA721703 |
|  | Prostate carcinoma | PRJNA219507 |

**Table S4: qRT-PCR primers in this study**

| **Primers for relative gene expression analysis** | | | |
| --- | --- | --- | --- |
| **Primer name** | **Gene** | **Sequence 5’–3’** | **Organism** |
| Robo3_ex1_Qb1 | *Robo3* | TCCAACTCCAGCGAGCTACT | Mouse |
| Robo3_ex2_Qb2 | *Robo3* | CTTCTGGCCCAACTCTTGAC | Mouse |
| Robo3_ex3_Qc1 | *Robo3* | TTGGTGACCTGGAAGAAAGG | Mouse |
| Robo3_ex4_Qc2 | *Robo3* | TGCCAGCATCACTCTTGAAC | Mouse |
| Robo3_ex25_Qd1 | *Robo3* | CCCTACAGAAGGGAGCACAG | Mouse |
| Robo3_ex26_Qd2 | *Robo3* | TCGCTCTAGGGAAGACATGC | Mouse |
| Robo3_Exon5_Qd1 | *Robo3* | AGATCCCCAGCCCAATCTAC | Mouse |
| Robo3_Exon6_Qd2 | *Robo3* | CCCTCGTCTTCAGAGCTCAC | Mouse |
| Robo3_Exon8_Qe1 | *Robo3* | GGGATGGTGGCTACTACGTG | Mouse |
| Robo3_Exon9_Qe2 | *Robo3* | CTGGTCCCTGGAGAATGATG | Mouse |
| Robo3_Exon11_Qf1 | *Robo3* | GGCCACAGCTACCTCCTATG | Mouse |
| Robo3_Exon12_Qf2 | *Robo3* | CAGGCCACTGATGGTGTAAG | Mouse |
| Robo3_Exon14_Qg1 | *Robo3* | GCTGAAAGCCCTTTTGTGAC | Mouse |
| Robo3_Exon15_Qg2 | *Robo3* | GAGGCTCCCAGGATACAGTG | Mouse |
| Robo3_Exon17_Qh1 | *Robo3* | GCTGCTACTCGGGTTCTGC | Mouse |
| Robo3_Exon18_Qh2 | *Robo3* | CCCTCTGAGTGTGGAAAGGA | Mouse |
| Robo3_Exon21_Qi1 | *Robo3* | CAAGCACCTGGAGCCAGTAT | Mouse |
| Robo3_Exon22_Qi2 | *Robo3* | CTTCTGGCCAGCTCAAAGAT | Mouse |
| Robo3_Exon27_Qj1 | *Robo3* | TCCAACAGCTCTCGAGGTTC | Mouse |
| Robo3_Exon28_Qj2 | *Robo3* | TGAAGGGTCATCTTGGTTCC | Mouse |
| Rplp0-Ex2_Qa1 | *Rplp0* | GATTCGGGATATGCTGTTGG | Mouse |
| Rplp0-Ex3_Qa2 | *Rplp0* | GCCTGGAAGAAGGAGGTCTT | Mouse |
| ABCA12_Qa1 | *ABCA12* | ATTCAGGCAGCAAAAACCAT | Human |
| ABCA12_Qa2 | *ABCA12* | TAGCCTCTGTGCCAGCTTCT | Human |
| ABCA2_Qa1 | *ABCA2* | GGTTCTTCAACCGCAACTTC | Human |
| ABCA2_Qa2 | *ABCA2* | TGCTCCATCTTGCTGAACAC | Human |
| ABCB1_Qa1 | *ABCB1* | AGGCCAACATACATGCCTTC | Human |
| ABCB1_Qa2 | *ABCB1* | AGCTATGGCAATGCGTTGTT | Human |
| ABCG1_Qa1 | *ABCG1* | ACTGCAGCATCGTGTACTGG | Human |
| ABCG1_Qa2 | *ABCG1* | GTCGAAGCTGACGAAGAACC | Human |
| ABCG2_Qa1 | *ABCG2* | GCAAATGCTGTCCTTTTGCT | Human |
| ABCG2_Qa2 | *ABCG2* | GGCCAATAAGGTGAGGCTATC | Human |
| ROBO3_Ex18/19_Qr1 | *ROBO3* | CTACACACCGGCAGTGTCCT | Human |
| ROBO3_Ex20_Qr2 | *ROBO3* | ATATCTGTCGTCCGGGTCAG | Human |
| ROBO3_Ex21_Qs1 | *ROBO3* | ACATCCCTCAGGAGATCTGG | Human |
| ROBO3_Ex22_Qs2 | *ROBO3* | TCCCCAGAAGCTTCACTTTG | Human |
| ROBO3_Ex23_Qt1 | *ROBO3* | TACACCCTCACCTCCTGACC | Human |
| ROBO3_Ex24_Qt1 | *ROBO3* | AGTGTATCCCAGCCCATGCT | Human |
| ROBO3_Ex24_Qt2 | *ROBO3* | ATGGGCTGGGATACACTGAG | Human |
| ROBO3_Ex25_Ql1 | *ROBO3* | AGTGTATCCCAGCCCATGCT | Human |
| ROBO3_Ex25_Qt2 | *ROBO3* | TGAAGTGGGGGAGTCATCTC | Human |
| ROBO3_Ex26_Ql2 | *ROBO3* | TGAAGTGGGGGAGTCATCTC | Human |
| ROBO3_Ex26_Qt1 | *ROBO3* | GTGCTCCACCCAGATGAAGA | Human |
| ROBO3_Ex27_Qm1 | *ROBO3* | CCAAAGGCCAGGACAGAAAC | Human |
| ROBO3_Ex27_Qt2 | *ROBO3* | GTTTCTGTCCTGGCCTTTGG | Human |
| ROBO3_Ex28_Qm2 | *ROBO3* | AAACCAATGGGCTTCAGCTA | Human |
| ROBO3_Ex3_Qb1 | *ROBO3* | AGGACGGTGCAAGACTCAAG | Human |
| ROBO3_Ex4_Qb2 | *ROBO3* | TTGGAGGCTACGCACACATA | Human |
| YAP1_Qa1 | *YAP1* | AACCCCAGATGACTTCCTGA | Human |
| YAP1_Qa2 | *YAP1* | CCAGGAATGGCTTCAAGGTA | Human |
| RPLP0_Qa1 | *RPLP0* | GATTGGCTACCCAACTGTTG | Human |
| RPLP0_Qa2 | *RPLP0* | CAGGGGCAGCAGCCACAAA | Human |

| **Primers and *ROBO3* DNA templates for absolute gene expression quantification (see Fig. 2C)** | | | |
| --- | --- | --- | --- |
| **DNA oligo name** | **Gene** | **Sequence 5’–3’** | **Organism** |
| ***ROBO3* DNA template** | | | |
| hROBO3_Ex1-2_DNA_Qa | *ROBO3* | CTCTTGGGCTTCAACTCCTCGCTGGCGGCGCTCAACCACACCCTGCTGCCTCCCGGCGATCCCTCTCTCAACGGGTCAAGGGTAGGACCGGAGGACGCTAT | human |
| hROBO3_Ex3-4_DNA_Qb | *ROBO3* | AGGACGGTGCAAGACTCAAGGAAGAGGAAGGAAGGATCACGATCCGTGGAGGGAAGCTGATGATGTCACATACACTCAAGAGCGATGCAGGCATGTATGTGTGCGTAGCCTCCAA | human |
| hROBO3_Ex5-6_DNA_Qc | *ROBO3* | TGCCCCTGTGACTTTCCTATGTGAGGTGAAGGGGGATCCCCCACCTCGTCTACGCTGGCGCAAGGAGGATGGGGAACTGCCCACAGGCAGGTATGAGATCCGGAGTGACCAC | human |
| hROBO3_Ex9-10_DNA_Qe | *ROBO3* | ATGGCCAACGGTACCCTGTACATCGCCAATGTGCAGGAGATGGACATGGGCTTCTACAGCTGCGTGGCCAAGAGTTCCACAGGGGAAG | human |
| hROBO3_Ex27-28_Qm | *ROBO3* | CAAAGGCCAGGACAGAAACGCCGAGAGGAACCAAGATGACCCTTGTTGGGGCATTGAGAATATCATGAGTGCCACGGGGA | human |
| **Primers for *ROBO3* DNA template** | | | |
| hROBO3_Ex1_Qa1 | *ROBO3* | CTCTTGGGCTTCAACTCCTC | human |
| hROBO3_Ex2_Qa2 | *ROBO3* | ATAGCGTCCTCCGGTCCTAC | human |
| hROBO3_Ex3_Qb1 | *ROBO3* | AGGACGGTGCAAGACTCAAG | human |
| hROBO3_Ex4_Qb2 | *ROBO3* | TTGGAGGCTACGCACACATA | human |
| hROBO3_Ex5_Qc1 | *ROBO3* | TGCCCCTGTGACTTTCCTAT | human |
| hROBO3_Ex6_Qc2 | *ROBO3* | GTGGTCACTCCGGATCTCAT | human |
| hROBO3_Ex9_Qe1 | *ROBO3* | ATGGCCAACGGTACCCTGTA | human |
| hROBO3_Ex10_Qe2 | *ROBO3* | CTTCCCCTGTGGAACTCTTGG | human |
| hROBO3_Ex27_Qm1 | *ROBO3* | CAAAGGCCAGGACAGAAACG | human |
| hROBO3_Ex28_Qm2 | *ROBO3* | TCCCCGTGGCACTCATGATA | human |

**Table S5: Primary antibodies**

| **Antibody** | **Species** | **Dilution** | **Source** | **Cat. Nr.** |
| --- | --- | --- | --- | --- |
| Actin | Goat polyclonal | 1:1,000 | Santa Cruz | sc-1616 |
| HSC70 | Goat polyclonal | 1:1,000 | Santa Cruz | sc-1059 |
| Robo3 | Rabbit polyclonal | 1:500 | Cusabio | CSB-PA291923 |
| SOX2 | Rabbit polyclonal | 1:1,000 | Millipore | Ab5603 |
| TEAD1 | Mouse polyclonal | 1:1,000 | BD Bioscience | BD-610922 |
| YAP1 | Rabbit polyclonal | 1:1,000 | Santa Cruz | sc-101199 |

**Table S6: Secondary antibodies**

| **Antibody** | **Conjugate** | **Dilution** | **Source** | **Cat. Nr.** |
| --- | --- | --- | --- | --- |
| Anti-mouse IgG | HRP | 1:10,000 | Santa Cruz | sc-2004 |
| Goat anti-rabbit IgG | HRP | 1:10,000 | Santa Cruz | sc-2005 |
| Donkey anti-goat IgG | HRP | 1:5,000 | Santa Cruz | sc-2020 |

**Table S7: siRNA sequences**

| **Gene** | **Sequence 5’-3’** | **Company** | **Cat. No** | **Organism** |
| --- | --- | --- | --- | --- |
| NT5 (Non-targeting control #5) | UGGUUUACAUGUCGACUAA | Dharmacon | D-001210-05 | Mouse, Human |
| Robo3 | GGGCAAGCCUGUACAAAUG  (Exon 22) GGAGCCAGCUGUAAUGGAA  (Exon 3 ) UCACAGAGGUAACCGCAAA  (Exon 11) GGGAGAACCCGUCAGGUAA  (Exon 25) | Dharmacon | M-055145-01 | Mouse |
| ROBO3 | CCAAAGGCCAGGACAGAAA  (Exon 27) | Dharmacon | D-026504-03 | Human |
| ROBO3 #2 | GGAACCAAGAUGACCCUUG  (Exon 28) | Thermo Scientific | AM16708 29905 | Human |
| TEAD1 | CACAAGACGUCAAGCCUUU GAAAGGUGGCUUAAAGGAA CGAUUUGUAUACCGAAUAA CCCAAUGUGUGAAUAUAUG | Dharmacon | M-012603-1 M-012603-2 M-012603-3 M-012603-4 | Human |

**Table S8: human ROBO3 exon definition file**

| **Chromosom** | **Start** | **End** | **Name** | **Score** | **Strand** |
| --- | --- | --- | --- | --- | --- |
| chr11 | 124865408 | 124865737 | NM_022370.3_exon_0_0_chr11_124865409_f | 0 | + |
| chr11 | 124868801 | 124869128 | NM_022370.3_exon_1_0_chr11_124868802_f | 0 | + |
| chr11 | 124869449 | 124869607 | NM_022370.3_exon_2_0_chr11_124869450_f | 0 | + |
| chr11 | 124869947 | 124870068 | NM_022370.3_exon_3_0_chr11_124869948_f | 0 | + |
| chr11 | 124870164 | 124870303 | NM_022370.3_exon_4_0_chr11_124870165_f | 0 | + |
| chr11 | 124870600 | 124870728 | NM_022370.3_exon_5_0_chr11_124870601_f | 0 | + |
| chr11 | 124871013 | 124871138 | NM_022370.3_exon_6_0_chr11_124871014_f | 0 | + |
| chr11 | 124872380 | 124872552 | NM_022370.3_exon_7_0_chr11_124872381_f | 0 | + |
| chr11 | 124872883 | 124873089 | NM_022370.3_exon_8_0_chr11_124872884_f | 0 | + |
| chr11 | 124873309 | 124873391 | NM_022370.3_exon_9_0_chr11_124873310_f | 0 | + |
| chr11 | 124873696 | 124873862 | NM_022370.3_exon_10_0_chr11_124873697_f | 0 | + |
| chr11 | 124874069 | 124874236 | NM_022370.3_exon_11_0_chr11_124874070_f | 0 | + |
| chr11 | 124874787 | 124874909 | NM_022370.3_exon_12_0_chr11_124874788_f | 0 | + |
| chr11 | 124875110 | 124875336 | NM_022370.3_exon_13_0_chr11_124875111_f | 0 | + |
| chr11 | 124875563 | 124875685 | NM_022370.3_exon_14_0_chr11_124875564_f | 0 | + |
| chr11 | 124875953 | 124876125 | NM_022370.3_exon_15_0_chr11_124875954_f | 0 | + |
| chr11 | 124876274 | 124876460 | NM_022370.3_exon_16_0_chr11_124876275_f | 0 | + |
| chr11 | 124877160 | 124877184 | NM_022370.3_exon_17_0_chr11_124877161_f | 0 | + |
| chr11 | 124877266 | 124877309 | NM_022370.3_exon_18_0_chr11_124877267_f | 0 | + |
| chr11 | 124877518 | 124877658 | NM_022370.3_exon_19_0_chr11_124877519_f | 0 | + |
| chr11 | 124877936 | 124878131 | NM_022370.3_exon_20_0_chr11_124877937_f | 0 | + |
| chr11 | 124878297 | 124878436 | NM_022370.3_exon_21_0_chr11_124878298_f | 0 | + |
| chr11 | 124878583 | 124878796 | NM_022370.3_exon_22_0_chr11_124878584_f | 0 | + |
| chr11 | 124879189 | 124879341 | NM_022370.3_exon_23_0_chr11_124879190_f | 0 | + |
| chr11 | 124879464 | 124879575 | NM_022370.3_exon_24_0_chr11_124879465_f | 0 | + |
| chr11 | 124879786 | 124879948 | NM_022370.3_exon_25_0_chr11_124879787_f | 0 | + |
| chr11 | 124880417 | 124880608 | NM_022370.3_exon_26_0_chr11_124880418_f | 0 | + |
| chr11 | 124881238 | 124881474 | NM_022370.3_exon_27_0_chr11_124881239_f | 0 | + |

**Figure S1**

**A)** Venn diagram of the commonly up- (left panel) and downregulated (right panel) genes in pG-2 and H8N8 cell lines after 48 hours CAF chemotherapy (logFC < -1 and padj < 0.05). **B)** Validation of the observed upregulation of *Robo3* transcription. pG-2 cells were treated with CAF or 5-FU for 48 h relative to vehicle by qRT-PCR. **C)** Read coverage of Robo3 in murine brain (publicly available mRNA-seq data). **D)** Visualisation of H3K27ac- and H3K27me3-occupancy at the *Robo3*-locus in pG-2 cells treated with CAF or vehicle for 48 hours. **E)** IGV tracks of H3K4me3, H3K27ac, and H3K27me3 at the Robo3-locus in MMTV-MYC mammary carcinomas (publicly available data). **F-G)** Western blot analysis of pG-2 cells (F) and H8N8 cells (G) treated with 48 hours CAF or 5-FU showing a 28 kDa band corresponding to the predicted molecular weight of a short ROBO3 isoform. Expression of full-length ROBO3 (expected at 150-200 kDa) is not detectable. (* unspecific bands) **H-I)** Validation of ROBO3 silencing by siROBO3 treatment in pG-2 cells exposed to 48 hours of CAF chemotherapy (left panel) via qRT-PCR (**H**) and western blot analysis (**I**). **J)** Proliferation assay of HCC1806 treated with siROBO3 over a duration of 5 days. Relative confluency measured by Celigo® and normalized to day 0. The Statistical analyses were performed on the under the curve. **K)** Quantification of *Robo3* expression via qRT-PCR in pG-2 cells grown in decreasing concentrations of FBS for 72 h. Data normalized to standard culture condition (10 % FBS). **L)** Quantification of tumor sphere formation assay of siControl or siRobo3 treated p-G2 cells under FBS starvation conditions. **B, H, J, K and L:** Error bars represent mean ± SEM of three biological replicates, unpaired t-test. * p < 0.05; ** p < 0.01; *** p < 0.001.

**Figure S2:**

Extracted fragment ion chromatograms of six unique murine Robo3 peptides. Fragment ion signals are exclusively detected after CAF treatment and not under control conditions. Left: Library spectra that were matched to the respective Robo3 peptides in the discovery approach. Right: Extracted fragment ion chromatograms for each Robo3 peptide after CAF treatment (three biological replicates depicted in the three left columns) and under control conditions (three biological replicates depicted in the three right columns). A dot product between the measured fragment ion signals and the spectral library match is indicated in the upper left or right corner of each chromatogram. The retention time at the peak apex and the mass deviation in parts per million is indicated above the peak. See the color legend below each spectrum. The retention time window includes unspecific co-eluting signals, which are present in both conditions and demonstrate that the same elution window is shown.

**Figure S3**

**A)** Relative *ROBO3s* mRNA expression assessed by qRT-PCR in different human cell lines. **B)** Occupancy tracks of Pol II, H3K4me3, H3K27ac, and H3K27me3 ChIP-seq at the *ROBO3* locus in HCC1806 cells from publically available data. These profiles uncover an active regulatory region in the proximity of exon 17 that could act as an alternative promoter for the expression of ROBO3s. **C-F)** Heatmap of *ROBO3* expression levels (RPKM) for each exon across publicly available RNA-seq data sets of TCGA patient data for several BC subtypes **(C),** different normal human tissues **(D)**, several other cancer types **(E)**, and GTEx human brain cortex patient data **(F)**. The datasets were sorted along the expression value of 23^rd^ exon.

**Figure S4**

**A)** BLAST protein sequence alignment and homology comparison of murine and human ROBO3s based on predicted ORFs. **B)** Extracted fragment ion chromatograms of the ROBO3 peptide after siRNA knockdown (left three columns) and after siRNA control treatment (right three columns), upper row for MDA-MB-231 cells and lower row for MDA-BM-468 cells. A dot product between the measured fragment ion signals and the spectral library match is indicated in the upper left corner of each chromatogram. The retention time at the peak apex and the mass deviation in parts per million is indicated above the peak. See the color legend below each spectrum. The retention time window includes unspecific co-eluting signals, which are present in both conditions and demonstrate that the same elution window is shown. **C)** Extracted fragment ion chromatograms of one unique human ROBO3 peptide. Fragment ion signals are less abundant after siRNA knockdown in comparison to a vehicle control treatment. Library spectrum that was matched to the respective Robo3 peptide in the discovery approach. **D)** Validation of siROBO3 KD in HCC1806 cells with siRNA ROBO3 #2 relative to siRNA control by qRT-PCR. **E)** Proliferation assay of HCC1806 treated with siROBO3 #2. **F-G)** Proliferation assays of MDA-MB-468 (**F**) and HCC-70 cells (**G**) treated with siROBO3. Relative confluency was measured by Celigo® and normalized to day 0. Quantification of proliferation differences by comparison of AUC. **D-G:** Error bars represent mean ± SEM of three biological replicates, unpaired t-test. * p < 0.05, ** p < 0.01, *** p < 0.001.

**Figure S5**

Extracted fragment ion chromatograms of three unique human ROBO3 peptides. Fragment ion signals are less abundant after siRNA knockdown in comparison to a vehicle control treatment. A stronger decrease is visible for HEK293T cells in comparison to Jurkat cells. Left: Library spectra that were matched to the respective ROBO3 peptides in the discovery approach. Right: Extracted fragment ion chromatograms for each Robo3 peptide after siRNA knockdown (three biological replicates depicted in the three left columns) and after vehicle control treatment (three biological replicates depicted in the three right columns), upper row for Jurkat cells and lower row for HEK293T cells. A dot product between the measured fragment ion signals and the spectral library match is indicated in the upper left corner of each chromatogram. The retention time at the peak apex and the mass deviation in parts per million is indicated above the peak. See the color legend below each spectrum. The retention time window includes unspecific co-eluting signals, which are present in both conditions and demonstrate that the same elution window is shown.

**Figure S6**

**A)** GSEA of the TCGA-BRCA BLBC data significantly enriched for “GO Positive regulation of axon extension” and “GO Actin filament bundle“ in the group of patients with high *ROBO3* levels. **B)** Tumor sphere formation assay of siControl and siROBO3 treated HCC1806 cells. Quantification of maximal tumorspheres diameter (left panel) and tumorsphere volume (right panel). **C)** Colony formation assay of siControl and siROBO3 treated HCC1806 cells (left panel) and the respective analysis of the median colony size per condition. **D-F)** GSEA of the TCGA-BRCA BLBC-patient data significantly enriched for “LIM Mammary stem cell UP” **(D)**, “Hallmark Epithelial-mesenchymal transition”and **(E)** as well as for “CORDENONSI YAP1 Conserved signature “**(F)** in the group of patients with high *ROBO3* levels. **G)** Gene expression analysis by qRT-PCR of *YAP1* after siROBO3 treatment in HCC1806 cells. **H)** Tumor sphere formation assay of siControl, siYAP1 or siTEAD1 treated HCC1806 cells. Quantification of maximal tumorspheres diameter (left panel) and tumorsphere volume (right panel). **I)** Proliferation assay of HCC1806 treated with siROBO3, siTEAD1 or a combination of both siRNAs. Relative confluency was measured by Celigo® and normalized to day 0. The statistical analyses were performed on the respective AUCs. **J-K)** Proliferation assays of HCC1806 treated with siROBO3 and/or high (1:4 dilution) **(J)** or sub-lethal (1:64 dilution) **(K)** concentrations of CAF. **L-M)** HCC1806 treated with siROBO3 and/or high (5 uM) **(L)** or sub-lethal (0.625 uM) **(M)** concentrations of Cisplatin (0.625 µM dilution). **B-C, G-M:** Error bars represent mean ± SEM of three biological replicates, unpaired t-test. * p < 0.05; ** p < 0.01; *** p < 0.001.
