# Supplementary material for: ROBO3s: a novel ROBO3 short isoform promoting breast cancer aggressiveness
Source: Cell Death Dis. 2022 Sep 3;13(9):762. doi: 10.1038/s41419-022-05197-7 (PMC9440919; doi:10.1038/s41419-022-05197-7)
Supplement: Supplementary file 4 — Full and uncropped western blots [file 41419_2022_5197_MOESM4_ESM.pdf]

Figure 5

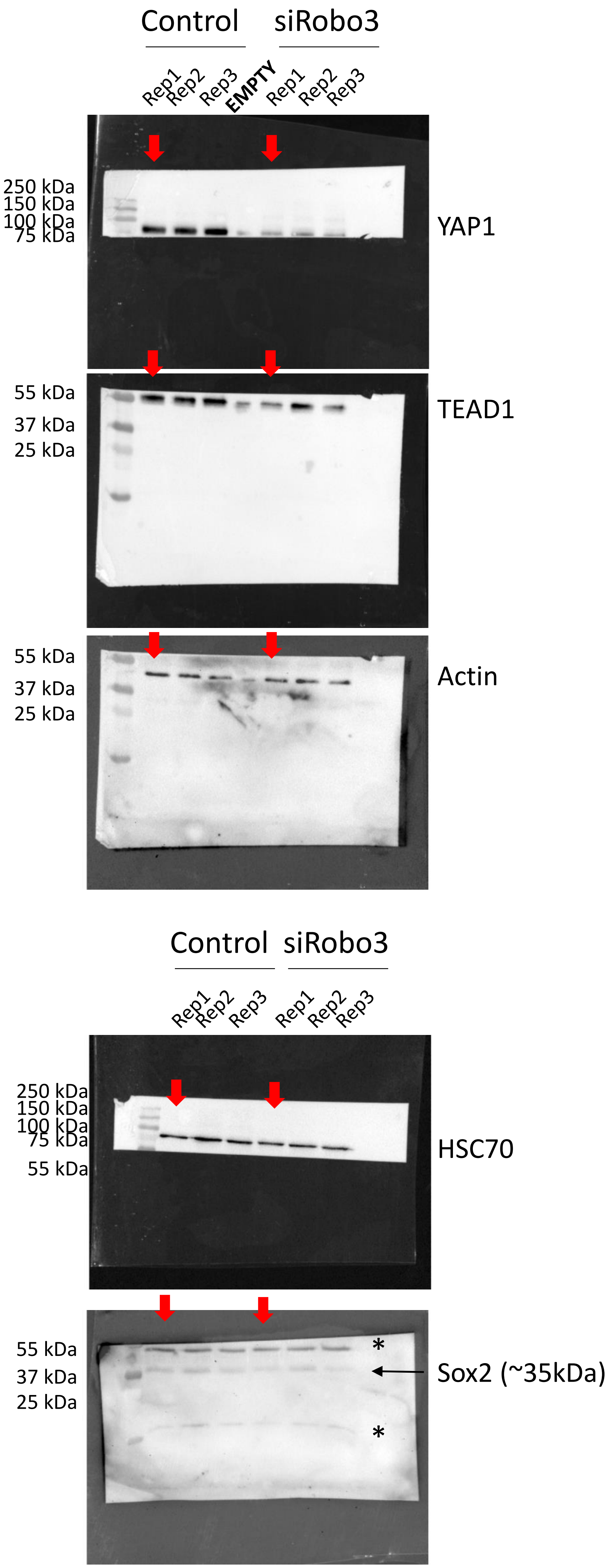

* Unspecific bands

Samples used in the figure

Figure S1F

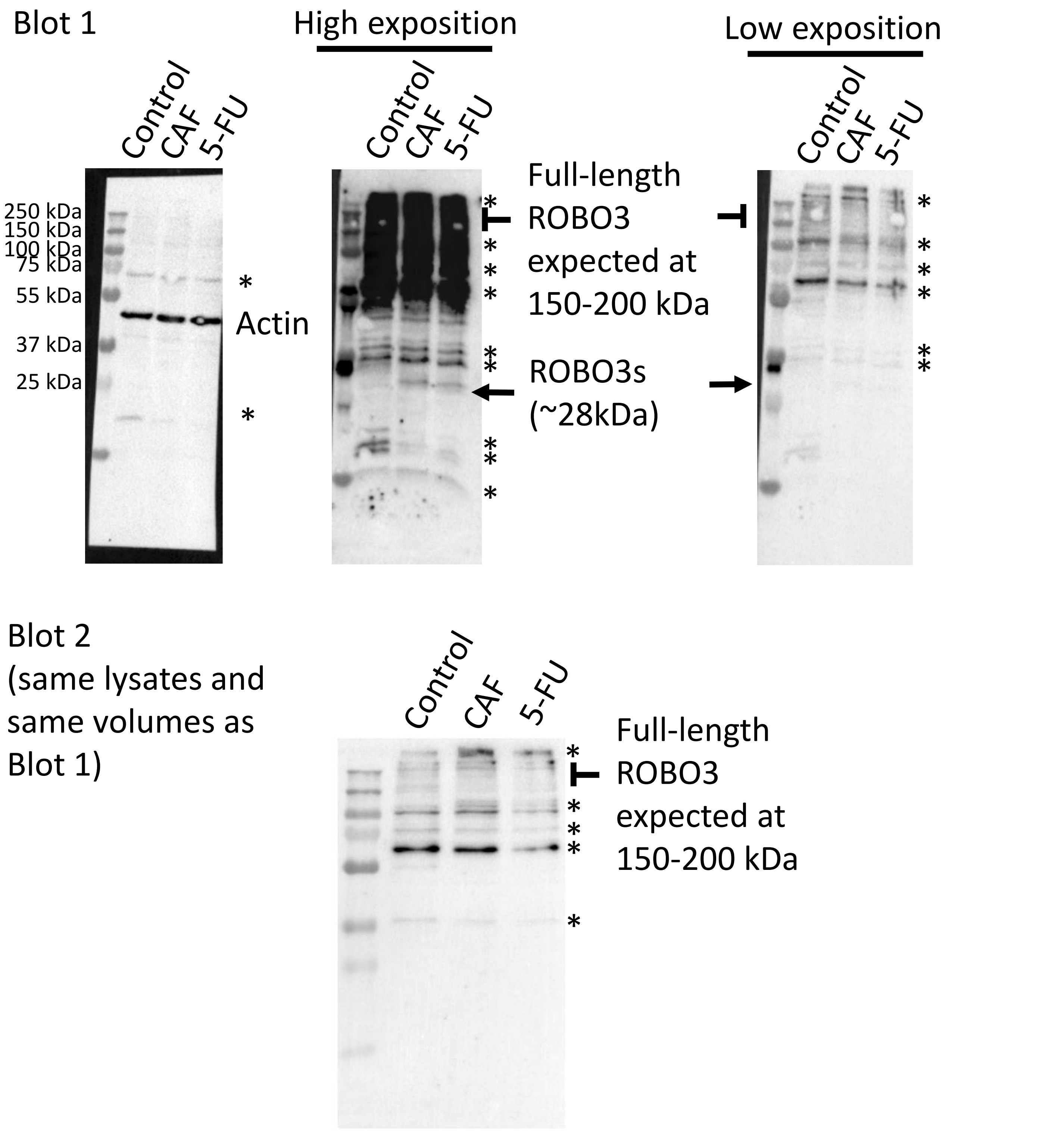

Figure S1I

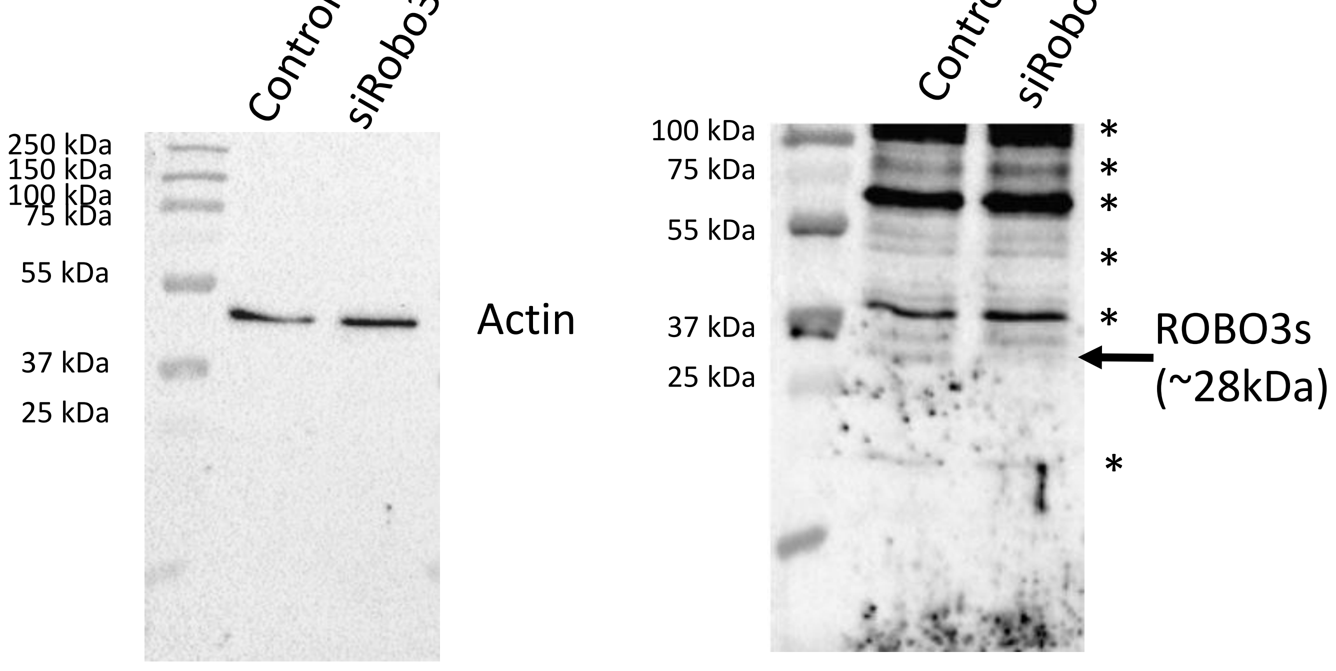

(* Unspecific Bands)

Figure S1G

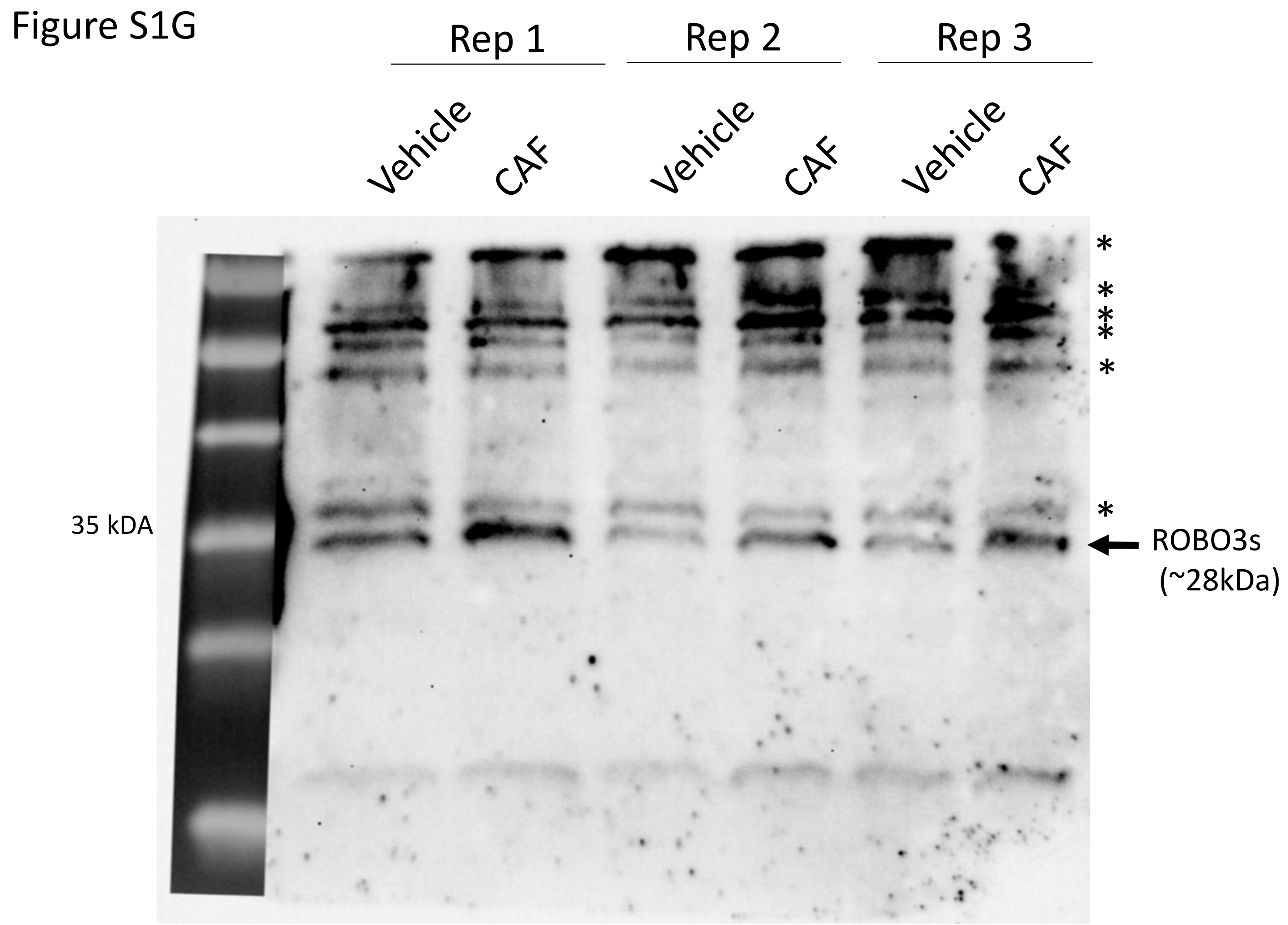

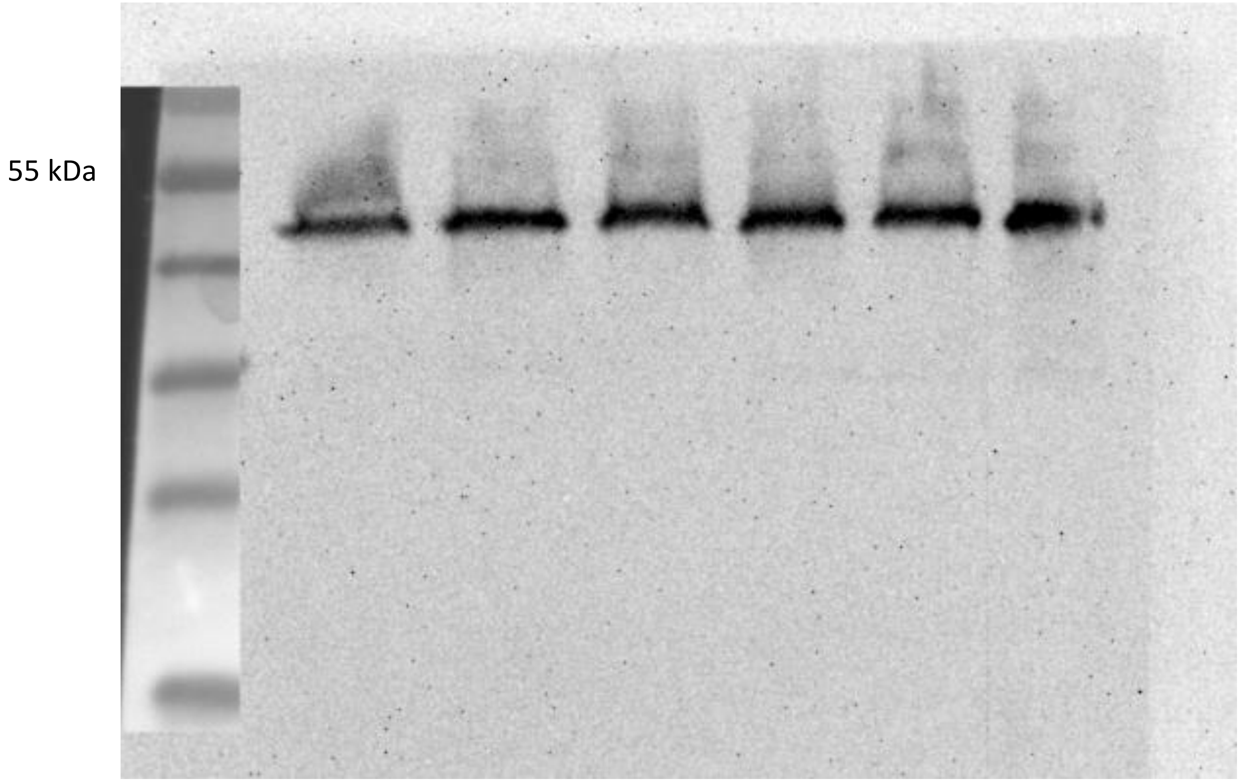

(* Unspecific Bands)
