# Supplementary figures and images for: ROBO3s: a novel ROBO3 short isoform promoting breast cancer aggressiveness
Source: Cell Death Dis. 2022 Sep 3;13(9):762. doi: 10.1038/s41419-022-05197-7 (PMC9440919; doi:10.1038/s41419-022-05197-7)

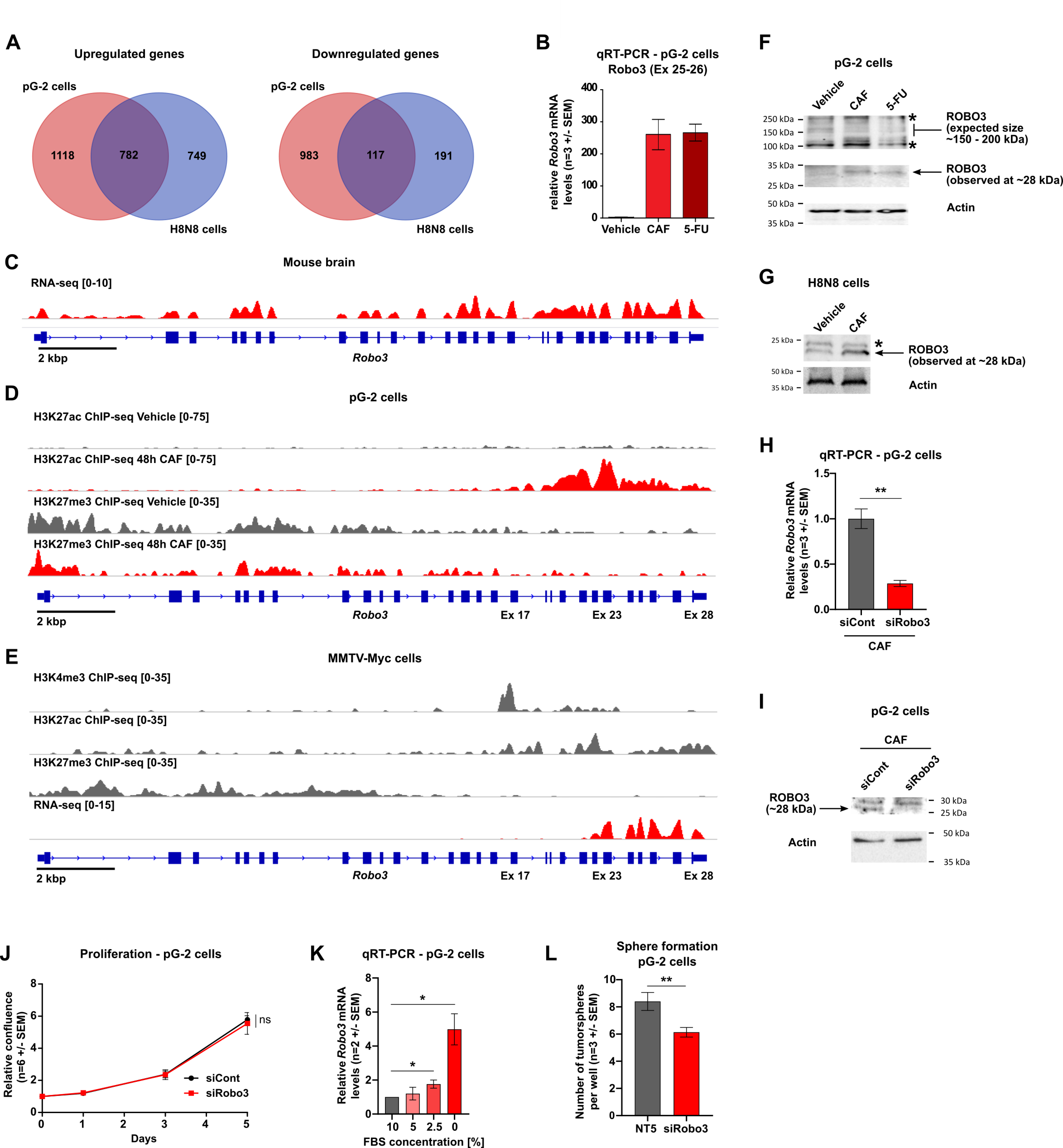

Supplement: Supplementary file 5 — Figure S1 [file 41419_2022_5197_MOESM5_ESM.tif]

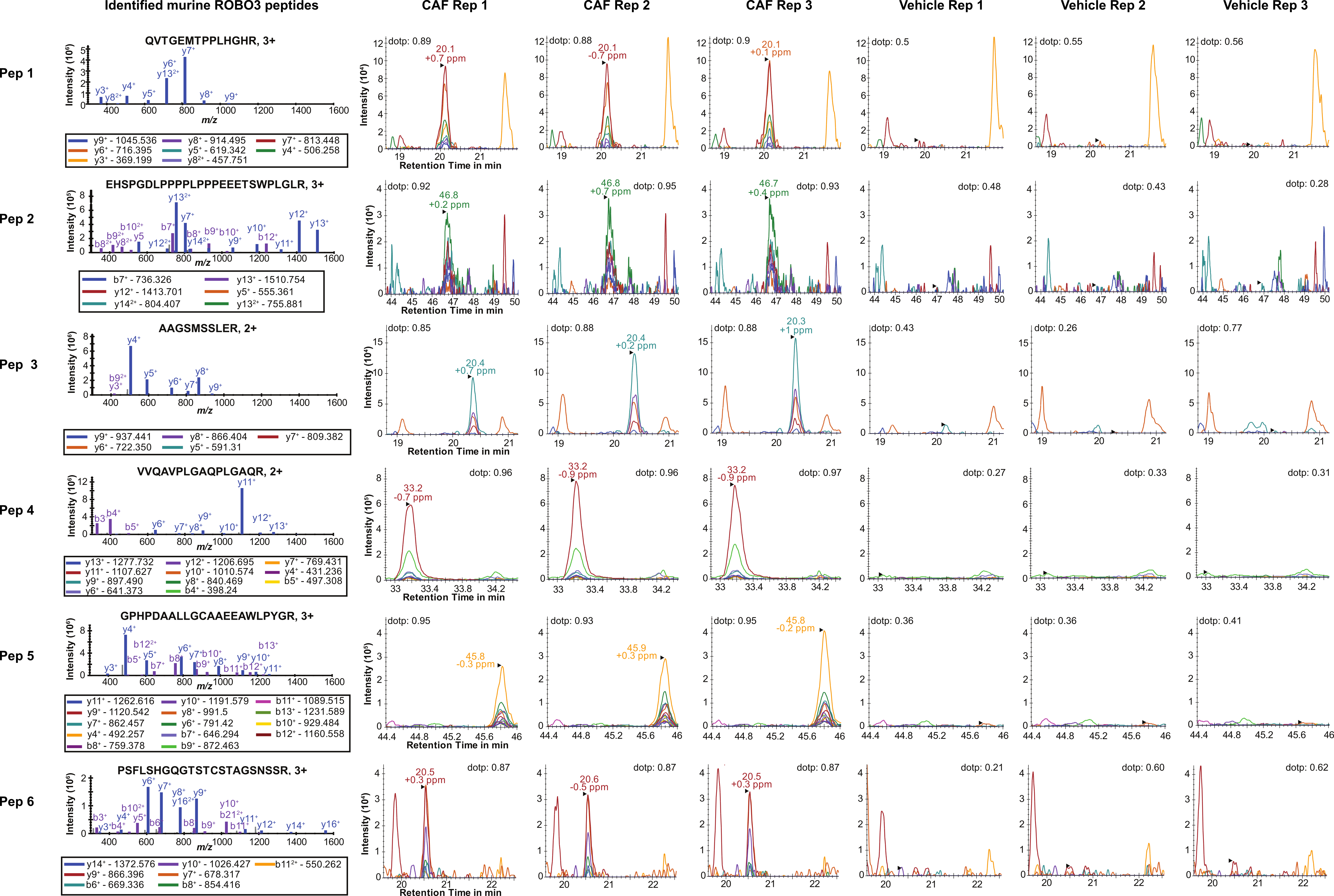

Supplement: Supplementary file 6 — Figure S2 [file 41419_2022_5197_MOESM6_ESM.tif]

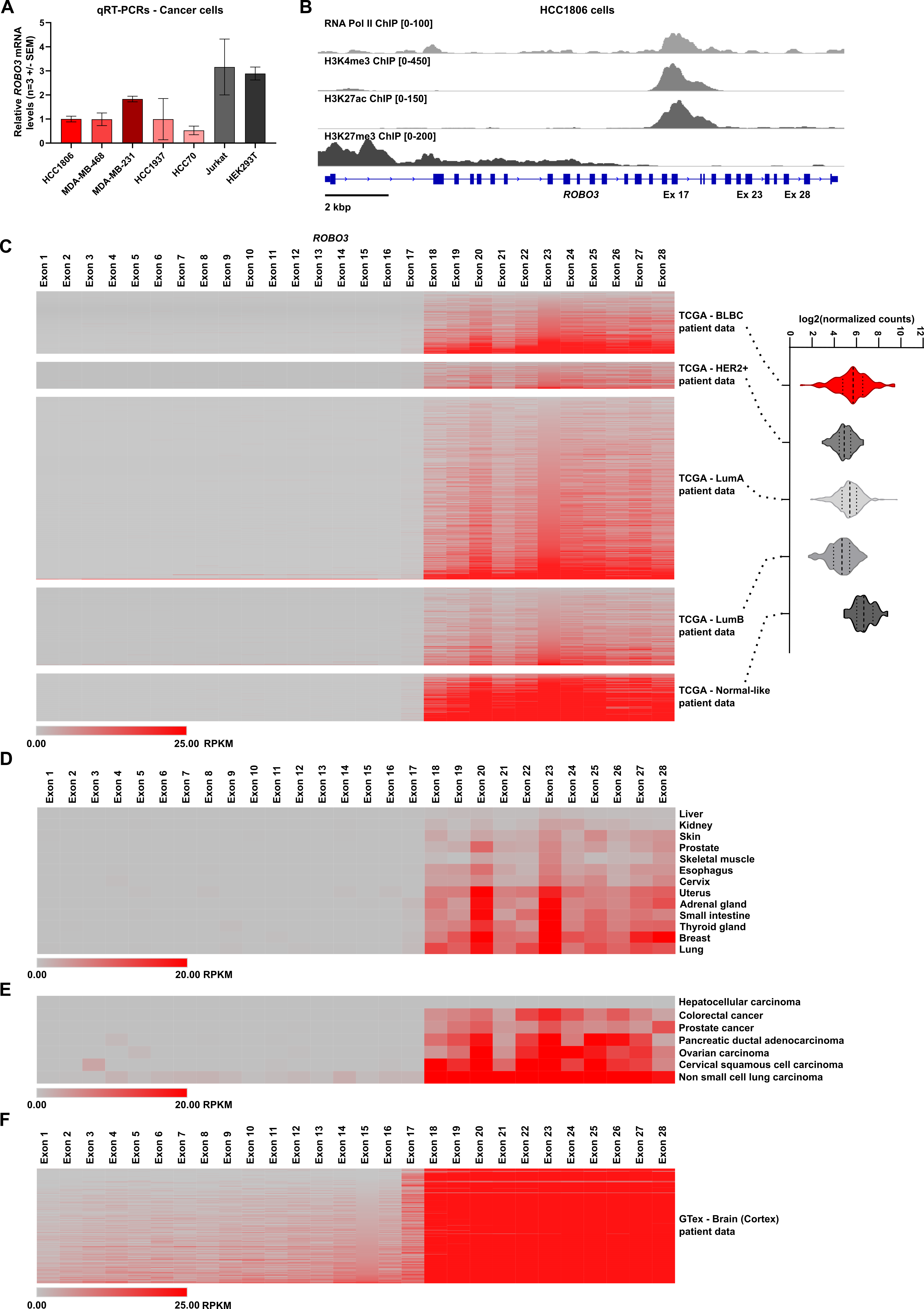

Supplement: Supplementary file 7 — Figure S3 [file 41419_2022_5197_MOESM7_ESM.tif]

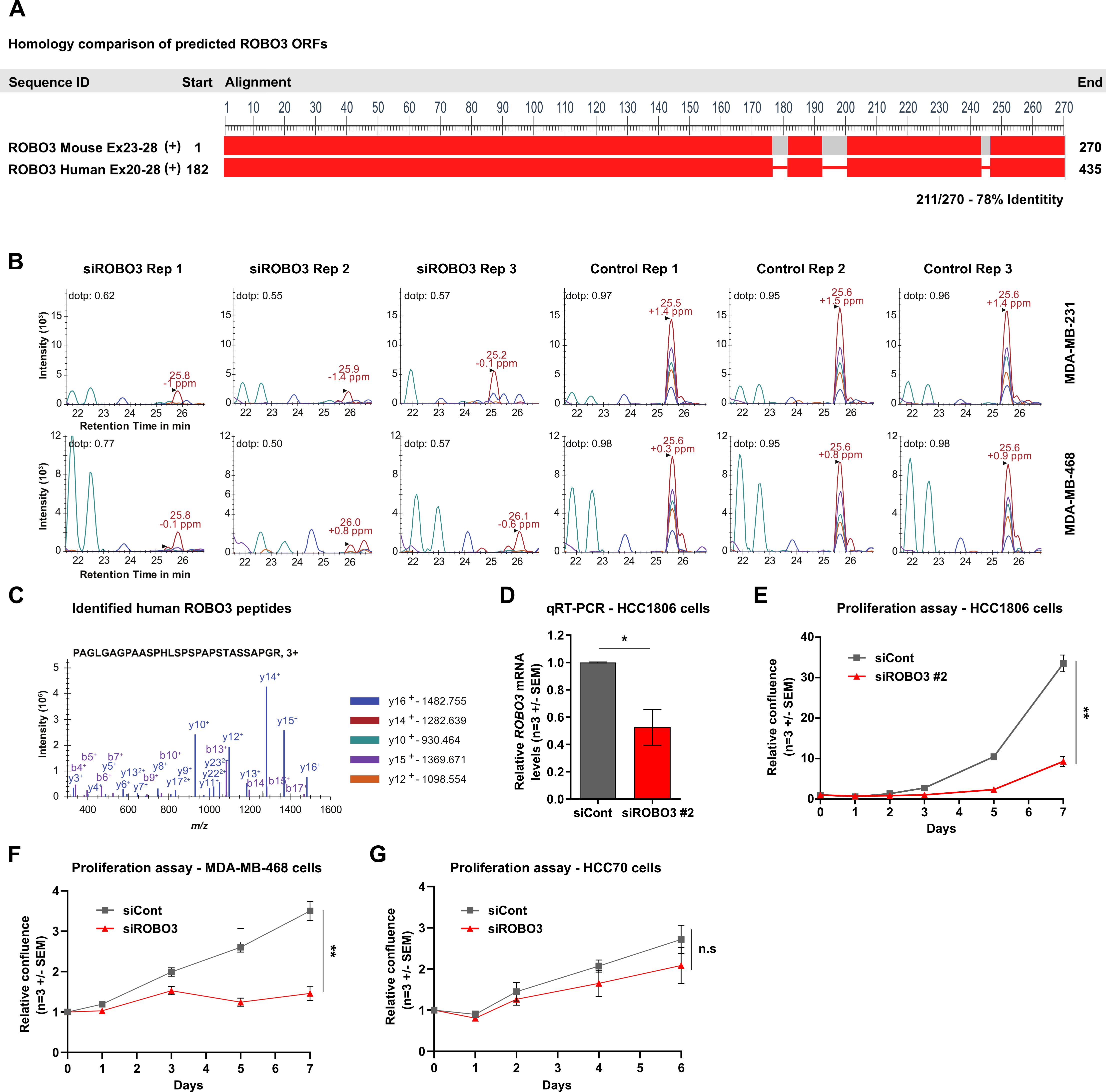

Supplement: Supplementary file 8 — Figure S4 [file 41419_2022_5197_MOESM8_ESM.tif]

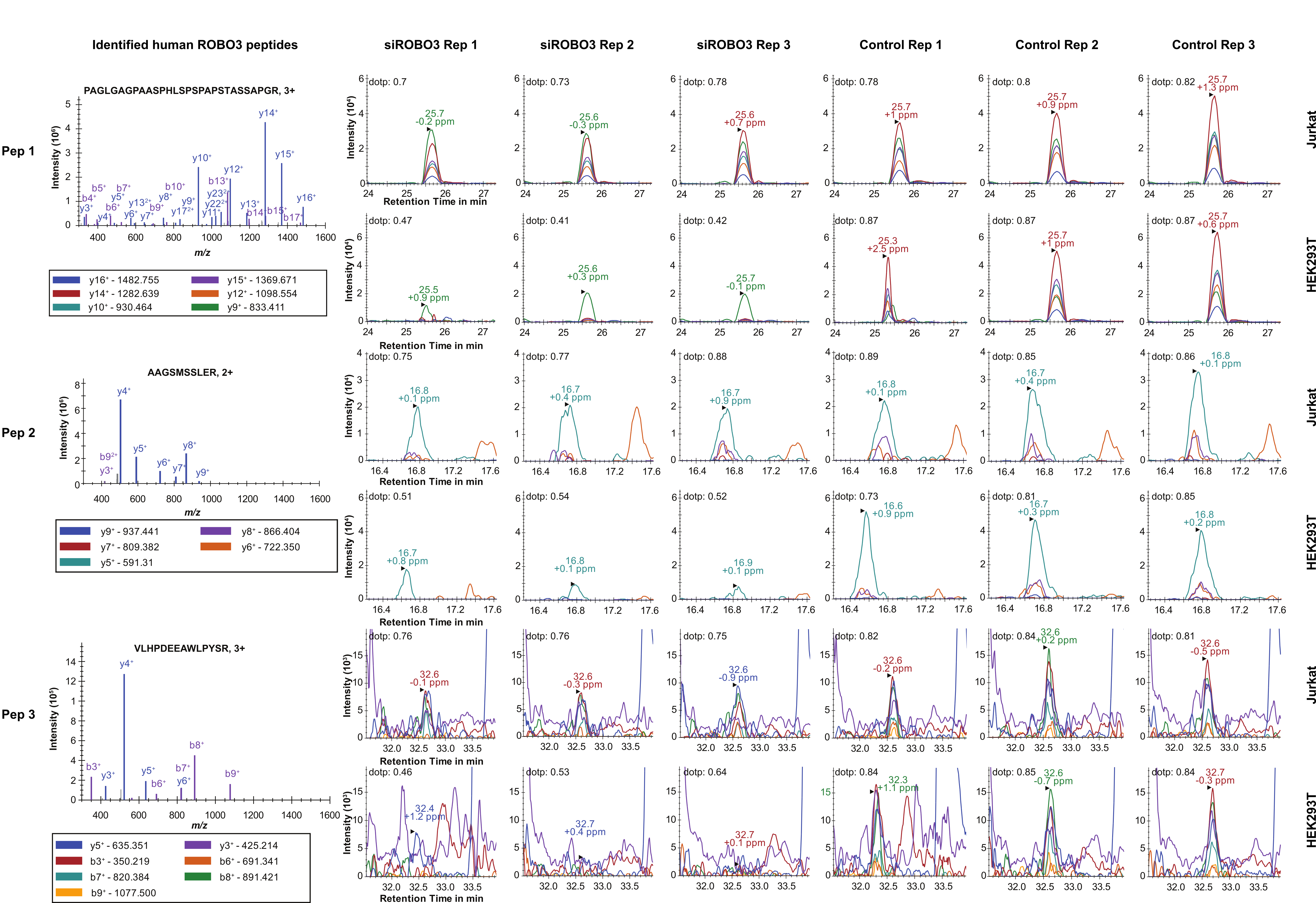

Supplement: Supplementary file 9 — Figure S5 [file 41419_2022_5197_MOESM9_ESM.tif]

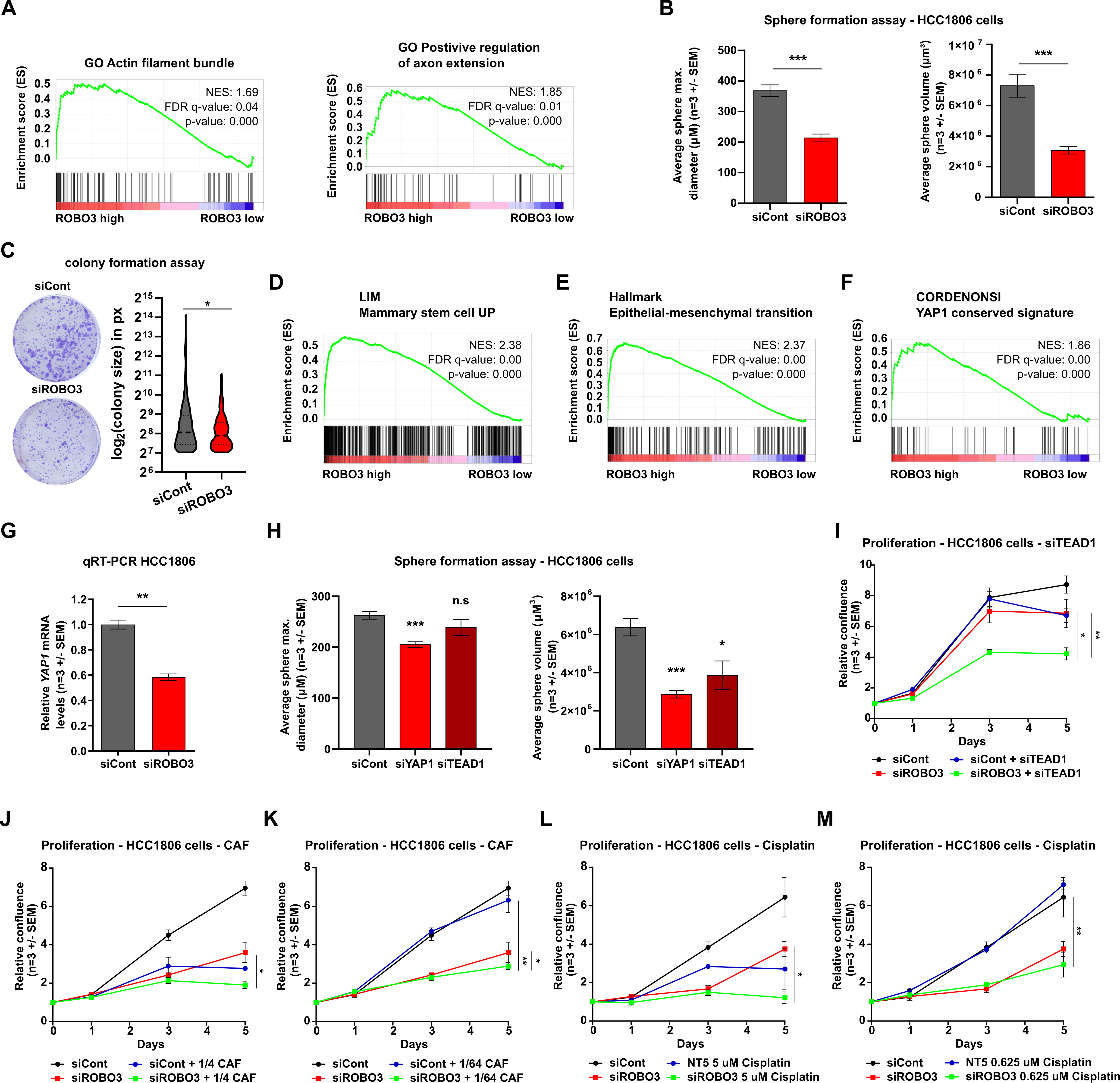

Supplement: Supplementary file 10 — Figure S6 [file 41419_2022_5197_MOESM10_ESM.tif]
